# Bone marrow endothelial dysfunction promotes myeloid cell expansion in cardiovascular disease

**DOI:** 10.1038/s44161-021-00002-8

**Published:** 2021-12-23

**Authors:** David Rohde, Katrien Vandoorne, I-Hsiu Lee, Jana Grune, Shuang Zhang, Cameron S. McAlpine, Maximilian J. Schloss, Ribhu Nayar, Gabriel Courties, Vanessa Frodermann, Gregory Wojtkiewicz, Lisa Honold, Qi Chen, Stephen Schmidt, Yoshiko Iwamoto, Yuan Sun, Sebastian Cremer, Friedrich F. Hoyer, Oriol Iborra-Egea, Christian Muñoz-Guijosa, Fei Ji, Bin Zhou, Ralf H. Adams, Joshua D. Wythe, Juan Hidalgo, Hideto Watanabe, Yookyung Jung, Anja M. van der Laan, Jan J. Piek, Youmna Kfoury, Pauline A. Désogère, Claudio Vinegoni, Partha Dutta, Ruslan I. Sadreyev, Peter Caravan, Antoni Bayes-Genis, Peter Libby, David T. Scadden, Charles P. Lin, Kamila Naxerova, Filip K. Swirski, Matthias Nahrendorf

**Affiliations:** 1 Center for Systems Biology, Massachusetts General Hospital and Harvard Medical School, Boston, MA, USA; 2 Department of Radiology, Massachusetts General Hospital and Harvard Medical School, Boston, MA, USA; 3 Department of Cardiology, Angiology and Pneumology, Heidelberg University Hospital, Heidelberg, Germany; 4 Biomedical Engineering Faculty, Technion-Israel Institute of Technology, Haifa, Israel; 5 Max Planck Institute for Molecular Biomedicine, Muenster, Germany; 6 Institut del Cor Germans Trias i Pujol, Barcelona, Spain; 7 Department of Genetics, Harvard Medical School, Boston, MA, USA; 8 Department of Molecular Biology, Massachusetts General Hospital, Boston, MA, USA; 9 State Key Laboratory of Cell Biology, CAS Center for Excellence in Molecular Cell Science, Institute of Biochemistry and Cell Biology, Chinese Academy of Sciences, Shanghai, China; 10 Cardiovascular Research Institute, Department of Molecular Physiology and Biophysics, Baylor College of Medicine, Houston, TX, USA; 11 Institute of Neurosciences and Department of Cellular Biology, Physiology and Immunology, Universitat Autonoma de Barcelona, Barcelona, Spain; 12 Institute for Molecular Science of Medicine, Aichi Medical University, Aichi, Japan; 13 Wellman Center for Photomedicine, Massachusetts General Hospital and Harvard Medical School, Boston, MA, USA; 14 Heart Center, Department of Cardiology, Amsterdam University Medical Center, University of Amsterdam, Amsterdam, the Netherlands; 15 Center for Regenerative Medicine and Cancer Center, Massachusetts General Hospital, Boston, MA, USA; 16 Department of Stem Cell and Regenerative Biology, Harvard University, Cambridge, MA, USA; 17 Martinos Center for Biomedical Imaging, Department of Radiology, Massachusetts General Hospital and Harvard Medical School, Charlestown, MA, USA; 18 Pittsburgh Heart, Lung, Blood and Vascular Medicine Institute, Division of Cardiology, Department of Medicine, University of Pittsburgh School of Medicine, Pittsburgh, PA, USA; 19 Department of Pathology, Massachusetts General Hospital and Harvard Medical School, Boston, MA, USA; 20 Division of Cardiovascular Medicine, Department of Medicine, Brigham and Women’s Hospital and Harvard Medical School, Boston, MA, USA; 21 Cardiovascular Research Center, Massachusetts General Hospital and Harvard Medical School, Boston, MA, USA; 22 Department of Internal Medicine I, University Hospital Wuerzburg, Wuerzburg, Germany; 23 These authors contributed equally: David Rohde, Katrien Vandoorne

## Abstract

Abnormal hematopoiesis advances cardiovascular disease by generating excess inflammatory leukocytes that attack the arteries and the heart. The bone marrow niche regulates hematopoietic stem cell proliferation and hence the systemic leukocyte pool, but whether cardiovascular disease affects the hematopoietic organ’s microvasculature is unknown. Here we show that hypertension, atherosclerosis and myocardial infarction (MI) instigate endothelial dysfunction, leakage, vascular fibrosis and angiogenesis in the bone marrow, altogether leading to overproduction of inflammatory myeloid cells and systemic leukocytosis. Limiting angiogenesis with endothelial deletion of *Vegfr2* (encoding vascular endothelial growth factor (VEGF) receptor 2) curbed emergency hematopoiesis after MI. We noted that bone marrow endothelial cells assumed inflammatory transcriptional phenotypes in all examined stages of cardiovascular disease. Endothelial deletion of *Il6* or *Vcan* (encoding versican), genes shown to be highly expressed in mice with atherosclerosis or MI, reduced hematopoiesis and systemic myeloid cell numbers in these conditions. Our findings establish that cardiovascular disease remodels the vascular bone marrow niche, stimulating hematopoiesis and production of inflammatory leukocytes.

Myeloid cells, innate immune guardians against infection, become protagonists of cardiovascular disease (CVD) in the presence of hyperlipidemia^1–3^. Increased myeloid cell abundance, that is, leukocytosis, is associated with worse cardiovascular outcomes in large clinical cohorts^4–7^. Leukocytes exert well-documented disease-propelling inflammatory functions in cardiovascular tissues^1–3^. Specifically, bone marrow-derived monocytes and neutrophils accumulate in atherosclerotic plaques in the arterial wall as well as in acutely ischemic and the chronically failing myocardium. Once recruited, leukocytes release pro-inflammatory cytokines and proteases that destabilize tissues and participate pivotally in the pathogenesis of atherosclerosis^1–3^ and heart failure^8,9^. Because myeloid cells such as monocytes circulate for a mere day after release from the bone marrow^10^, examining the pathways that instigate their overproduction in CVD may provide a key to curbing cardiovascular inflammation and morbidity. We here examined how the bone marrow vasculature adapts to three milestone conditions that typically develop consecutively in patients with CVD: (1) arterial hypertension, (2) atherosclerosis and (3) acute MI. A prototypical disease trajectory may begin with long-standing increased blood pressure and hyperlipidemia, moving on to chronic formation of inflammatory atherosclerotic plaques in the arteries’ intima and finally culminating in MI as a thrombotic complication of atherosclerosis, an acute event that is among the leading causes of death worldwide.

Myeloid cells derive from hematopoietic stem and progenitor cells (HSPCs) that reside in the hematopoietic bone marrow niche near abluminal surfaces of the microvascular endothelium^11^. Bone marrow endothelial cells shape hematopoiesis via secreted and cell surface-associated signals received by HSPCs^12–14^. In addition, endothelial cells form a barrier that regulates exchange with the blood pool and cell traffic in and out of the marrow. Such vascular functions modulate the numbers and types of circulating leukocytes in the steady state and during infection^15–17^.

Given the importance of endothelial niche cells for regulation of hematopoiesis^17,18^, we here investigated how CVD affects these cells. The bone marrow vasculature encounters the same stimuli that instigate CVD elsewhere. Countless such factors emerged from vascular biology research focusing on the heart and brain, for instance, (1) aberrant mechanical forces, for example, increased blood pressure altering vascular diameter, cell activation states, matrix deposition and vasomotor tone, (2) pathological metabolite blood content, for example, hyperlipidemia resulting in lipid deposits in the arterial wall, (3) reduced blood oxygen levels leading to angiogenesis or hypoxic cell death, (4) increased levels of circulating hormones, for example, angiotensin II (Ang-II), causing vasoconstriction, and (5) higher blood cytokine levels, for example, interleukins changing endothelial phenotypes. We examined whether these pathologies extend to the bone marrow, testing the hypothesis that pathways active in hypertension, atherosclerosis and acute MI affect vascular morphology and endothelial cell phenotypes in this hematopoietic organ. We further postulated that such alterations give rise to the overproduction of inflammatory myeloid cells and systemic leukocytosis, which is well documented to promote CVD^19–28^. Testing these hypotheses, we observed profound morphological, functional and molecular adaptations of the bone marrow vasculature to hypertension, atherosclerosis or MI. The variations were characteristic for each examined condition but ultimately converged on expanding leukocytosis. Conceptually, our data add a missing link to the vicious inflammatory cycle that propels CVDs by identifying bone marrow vascular pathologies that augment the supply of leukocytes implicated in these conditions.

## Results

### HSPCs proliferate more in human CVD.

**Fig. 1 |:**
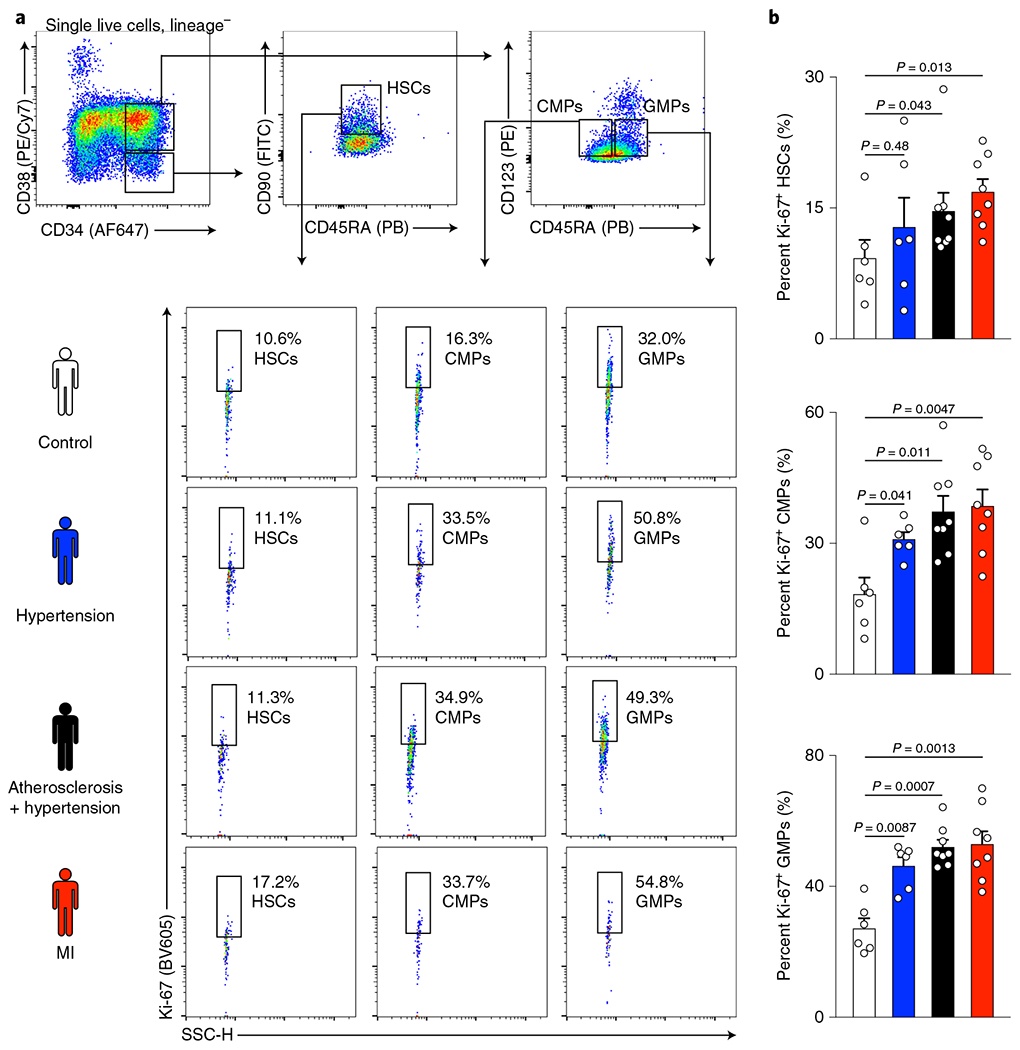
Increased bone marrow myelopoiesis in human CVD. **a**, Gating on human bone marrow HSPCs and their proliferative state by Ki-67 staining. Flow cytometry gating was defined by the use of fluorescence-minus-one controls for each sample. **b**, Quantification of Ki-67^+^ proliferation rates in phenotypic HSCs, CMPs and GMPs (*n* = 6 healthy control participants, *n* = 6 patients with arterial hypertension, *n* = 8 patients with atherosclerosis and arterial hypertension, *n* = 8 patients on day 3 to day 7 after acute MI; two-tailed Mann-Whitney tests between controls and each patient cohort without multiple-comparison correction). Data are displayed as mean ± s.e.m.

We formulated the overarching hypothesis that CVD affects key hematopoietic niche cells (bone marrow endothelial cells) and their interactions with hematopoietic progenitors. Given that endothelial cells and the vascular niche may increase progenitor proliferation by changing the blood stem cells’ microenvironment, we reasoned that dysregulation of the bone marrow vasculature would heighten leukocytosis. To determine which HSPCs are affected, we examined HSPCs in patients with arterial hypertension only, both hypertension and atherosclerosis or acute MI. Employing flow cytometry^29^ with intracellular staining for Ki-67, we found increased proliferation rates of undifferentiated hematopoietic stem cells (HSCs) in all three patient cohorts (Fig. 1). The fraction of actively cycling cells also expanded in more downstream common myeloid progenitors (CMPs) and granulocyte-monocyte progenitors (GMPs) (Fig. 1a,b). These results, which match recently obtained data in atherosclerosis^30^, indicated that the studied cardiovascular conditions affect the entire hematopoietic hierarchy, ranging from upstream HSCs to downstream myeloid progenitors. Going forward, we therefore examined how CVD-induced niche abnormalities influence progenitor cells affected in patients with CVD, using mice with the matching experimental milestone conditions of CVD: hypertension, atherosclerosis and acute MI.

### HSCs expand in hypertension.

**Extended Data Fig. 1 |:**
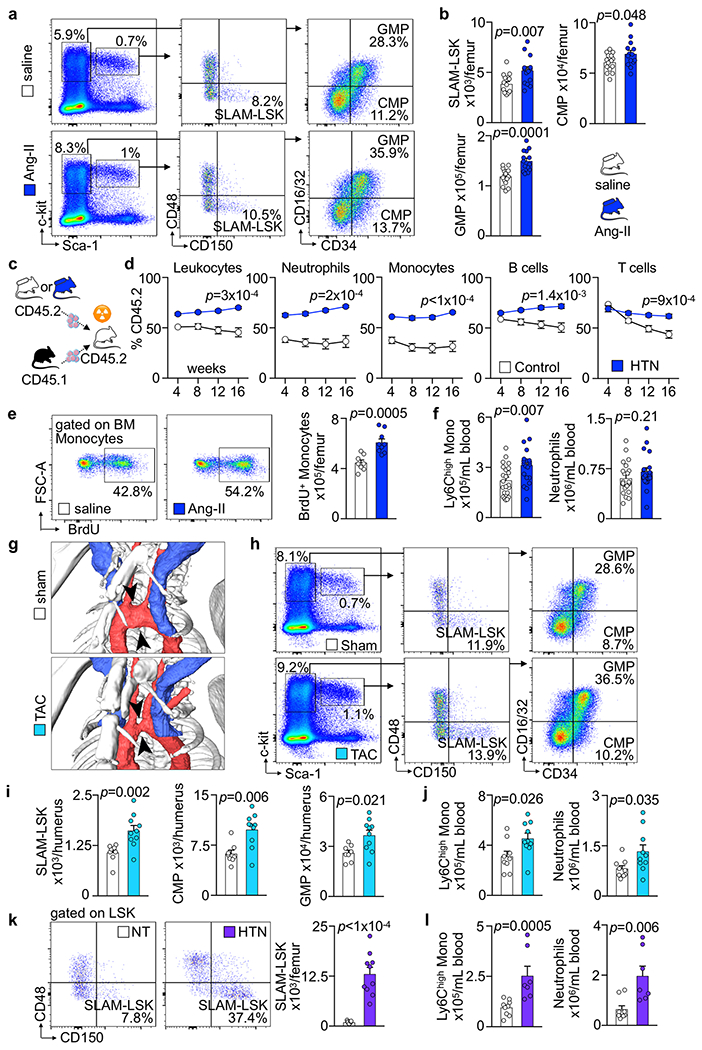
Arterial hypertension activates murine hematopoiesis. **a, b**, Flow plots (**a**) and numbers (**b**) of SLAM-LSK, CMP and GMP per femur in mice with saline or Angiotensin II (Ang-II) minipumps (saline n=15 mice, Ang-II n=14, two-tailed Welch’s t-test). **c**, Experimental outline for (**d**). 5×10^5^ bone marrow mononuclear cells (BMNC) were isolated from wild type mice (CD45.2) treated with saline or Angiotensin II (Ang-II) minipumps for 8 weeks and mixed with equal numbers of cells isolated from naive CD45.1 mice before transplantation into lethally irradiated CD45.2 recipients. **d**, Blood leukocyte chimerism after bone marrow transplantation (n=10 recipient mice per group, two-tailed Welch’s t-test for %CD45.2 in week 16). **e**, Flow plots and quantification of BrdU^+^ bone marrow monocytes (saline n=8 mice, Ang-II n=9, two-tailed Welch’s t-test). **f**, Ly6C^high^ monocytes and neutrophil numbers in the blood (saline n=22, Ang-II n=20, two-tailed Welch’s t-test). **g**, In vivo 3D computed tomography (CT) angiography. Arrowheads indicate transverse aortic constriction (TAC). **h**, **i**, Flow plots (**h**) and quantification (**i**) of SLAM-LSK, CMP and GMP in the right hypertensive humerus of sham- or TAC-operated animals 4 weeks after surgery (sham n=8 mice, TAC n=10, two-tailed Mann-Whitney test). **j**, Ly6C^high^ monocytes and neutrophils in blood (sham n=9, TAC n=10, two-tailed Welch’s t-test). **k**, Flow plots and quantification of SLAM-LSK in the femur of normotensive BPN/3J (NT) or hypertensive BPH/2J (HT) mice (NT n=13, HT n=10, two-tailed Welch’s t-test). **l**, Enumeration of blood Ly6C^high^ monocytes and neutrophils (NT n=9, HT n=7, two-tailed Mann-Whitney test). Data are displayed as mean±SEM.

In contrast to hyperlipidemia^22^, atherosclerosis^31^ and acute MI^32^, it was less clear whether hypertension affected HSPC proliferation in mice. To test whether hypertension, a risk factor preceding atherosclerosis and MI in patients, alters hematopoiesis, we profiled mouse HSPC numbers, their proliferation and the bone marrow’s myeloid cell output across three different modes of hypertension induction. First, we generated hypertension with Ang-II, which increased the number of bone marrow Lineage– c-kit+ Sca-1+ CD150+ CD48− (SLAM–LSK), CMPs and GMPs (Extended Data Fig. 1a,b). Competitive bone marrow transplantation from normotensive or hypertensive donors revealed a higher blood chimerism for leukocytes originating from hypertensive mice (Extended Data Fig. 1c,d). In accordance with increased monocyte production in the bone marrow of mice implanted with minipumps releasing Ang-II (Extended Data Fig. 1e), we found increased inflammatory Ly6C^hi^ monocytes and a trend toward more neutrophils in the blood (Extended Data Fig. 1f). Second, hypertension produced by aortic constriction between the brachiocephalic and left common carotid arteries (Extended Data Fig. 1g) increased SLAM–LSK, CMP and GMP numbers in the bone marrow of the hypertensive right humerus (Extended Data Fig. 1h,i) and numbers of Ly6C^hi^ monocytes and neutrophils in the blood (Extended Data Fig. 1j). Third, genetically induced neurogenic hypertension in BPH/2J mice^33^ produced similar results (Extended Data Fig. 1k,l). We conclude that, similar to atherosclerosis and after MI, hematopoiesis increases in hypertensive humans and in mice. In our subsequent studies, we therefore investigated how these three CVD milestones influence the vascular niche phenotype and function in the bone marrow.

### The hematopoietic niche during incipient disease.

**Extended Data Fig. 2 |:**
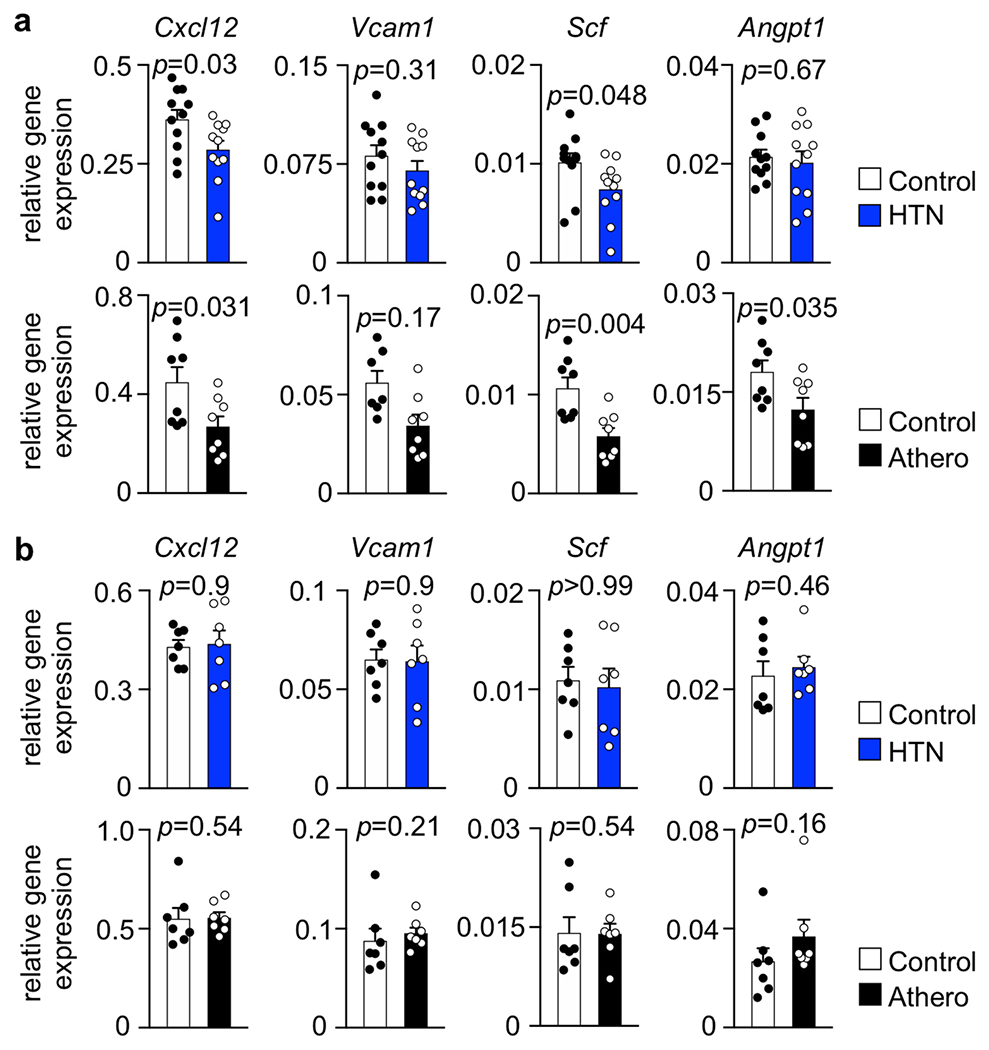
Bone marrow niche factor expression in mice with arterial hypertension and atherosclerosis. **a**, Quantification of niche factor expression in whole bone marrow of mice 2 weeks after initiating Ang-II treatment or starting atherogenic diet in *Apoe*^−/−^ mice (saline n=11 mice, Ang-II n=11, wild type n=8, *Apoe*^−/−^ n=8, two-tailed Welch’s t-test). **b**, Quantification of niche factor expression in whole bone marrow of mice 8 weeks after initiating Ang-II treatment or starting atherogenic diet in *Apoe*^−/−^ mice (n=7 mice per group, two-tailed Mann-Whitney test). Data are displayed as mean±SEM.

The bone marrow stem cell niche is formed by different cell types organized around arterioles and sinusoids. An ensemble of niche cells that, in addition to endothelial cells, also includes mesenchymal stromal cells, osteoblasts and macrophages^34,35^ regulates HSPC activity via signals known as niche factors^17,36^. We determined the expression of *Cxcl12, Vcam1, Scf* (*Kitl*) and *Angpt1*, thus sampling the canonical factors supplied by various niche cells to promote HSPC retention and quiescence in steady state^17,36^ that decline after acute MI^37^ in mice with hypertension or atherosclerosis. Whole-bone marrow expression of retention and maintenance factors decreased 2 weeks after implantation of Ang-II-releasing minipumps and 2 weeks after switching *Apoe*^−/−^ mice to an atherogenic diet (Extended Data Fig. 2a). At later time points after disease induction, specifically 2 months after the onset of hypertension or the beginning of atherogenic diet consumption, levels of these niche factors no longer differed from those of controls (Extended Data Fig. 2b), stimulating us to search for other pathways that chronically augment leukocytosis in CVD.

### Bone marrow vascular anatomy changes in CVD.

**Extended Data Fig. 3 |:**
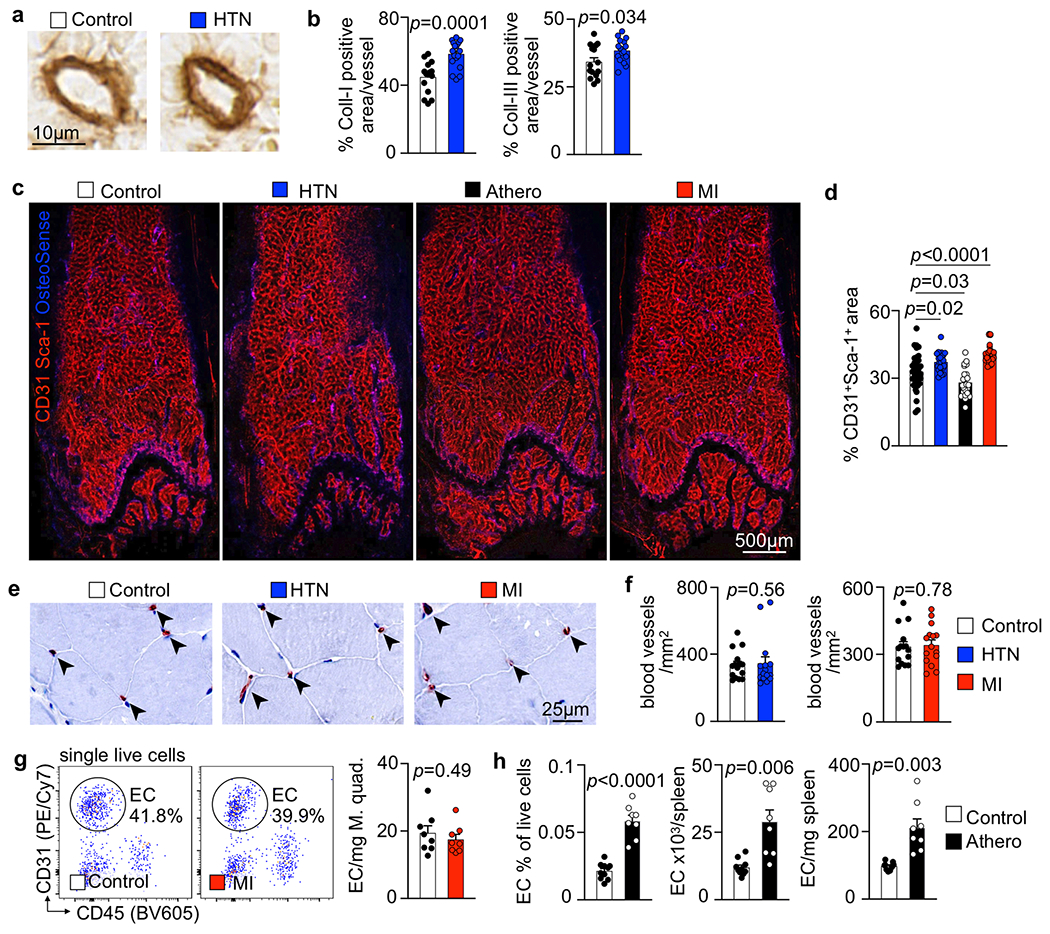
Cardiovascular disease-induced structural changes in bone marrow vasculature. **a, b**, Immunohistochemical staining for collagen III (**a**) and quantification of collagen I (Coll-I) and collagen III (Coll-III) (**b**) in hypertensive Ang-II-treated mice (Coll-I: saline n=15, Ang-II n=18; Coll-III: saline n=15, Ang-II n=16; two-tailed Welch’s t-test). **c**, Representative confocal immunofluorescence microcopy (tile scans) displaying the distribution of CD31^+^Sca-1^+^ blood vessels across the distal femoral bone marrow of naive controls, mice with Angiotensin II induced arterial hypertension (HTN), atherosclerotic *Apoe*^−/−^ mice on Western diet (Athero), and mice 6 days after myocardial infarction (MI).**d**, Quantification of blood vessel area (n=6 fields of view from n=6 controls and n=4 HTN/Athero/MI mice, one-way ANOVA with Dunnett’s T3 post-test).**e**, CD31 immunohistochemistry obtained in M. quadriceps femoris from naive controls, mice with Angiotensin II-induced arterial hypertension (Ang-II) and mice on day 6 after MI. Arrowheads indicate CD31^+^ capillaries. **g**, Flow plots and quantification of endothelial cells in the M. quadriceps femoris of controls and mice 6 days post myocardial infarction (n=8 mice per group, two-tailed Welch’s t-test). **h**, Quantification of endothelial cells in the spleen of WT and Apoe^−/−^ mice with atherosclerosis by flow cytometry (n=10 WT mice, n=8 *Apoe*^−/−^, two-tailed Welch’s t-test). Data are displayed as mean±SEM.

**Fig. 2 |:**
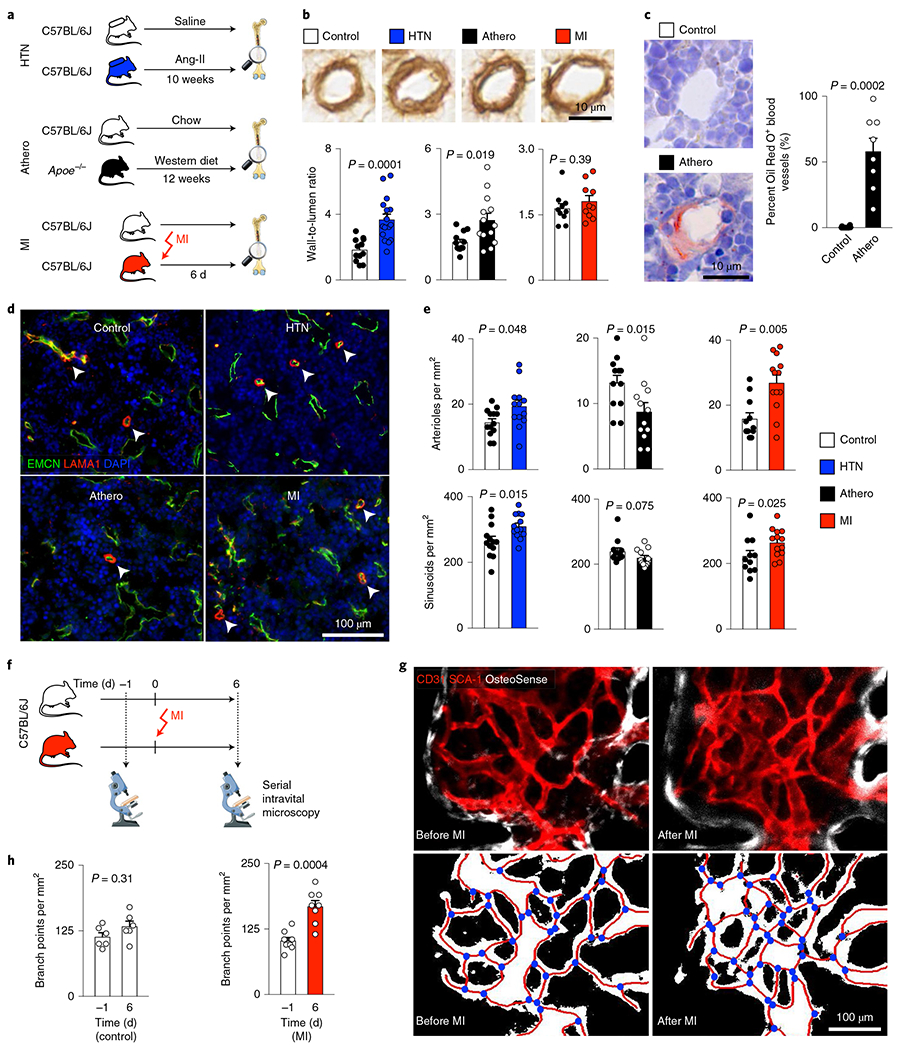
Arterial hypertension, atherosclerosis and MI alter the bone marrow vasculature. **a**, Experimental outline for mouse models of Ang-II-induced arterial hypertension (HTN), atherosclerosis (athero) and acute MI. **b**, Immunohistochemical staining for collagen IV and wall-to-lumen ratios of femoral bone marrow arterioles (*n* = 12 saline-treated mice, *n* = 16 with Ang-II (hypertension); *n* = 12 wild-type mice, *n* = 13 *Apoe*^−/−^ mice on a western diet (atherosclerosis); *n* = 10 controls, *n* = 10 with MI (day 6); two-tailed Mann-Whitney test). **c**, Staining and quantification of Oil Red O^+^ bone marrow blood vessels in wild-type (control) and *Apoe*^−/−^ mice (atherosclerosis, *n* = 8 mice per group, two-tailed Mann-Whitney test). Images (**d**) and quantification (**e**) of endomucin (EMCN) immunofluorescence for sinusoids and laminin α1 (LAMA1) for arterioles in the femur metaphysis (*n* = 13 mice per group for hypertension, *n* = 12 for atherosclerosis, *n* = 11 controls and *n* = 13 for MI; two-tailed Mann-Whitney test). DAPI, 4′,6-diamidino-2-phenylindole. Experimental outline (**f**), images (**g**) and quantification (**h**) of vascular branch points by serial intravital microscopy of the skull bone marrow 1 d before and 6d after MI (*n* = 8 mice per group, two-tailed paired *t*-test). SCA-1, stem cell antigen 1. Data are displayed as mean ± s.e.m.

Structural and functional vascular changes, which are central to the pathogenesis of hypertension, atherosclerosis and ischemia, occur system wide. Yet, how these conditions affect bone marrow vasculature, which regulates HSPC activity and leukocyte trafficking^12–14^, is unknown. To test the hypothesis that CVD-related mechanisms induce vascular changes in the bone marrow, we studied the femur and skull vasculature in mice with hypertension, atherosclerosis and acute MI. We detected increased femoral arteriolar wall thickness and collagen deposition after inducing the chronic conditions hypertension or atherosclerosis but not acutely after MI (Fig. 2a,b). Bone marrow arterioles of hypertensive mice displayed elevated staining for collagens I and III (Extended Data Fig. 3a,b). In *Apoe*^−/−^ mice with atherosclerosis, Oil Red O^+^ lipid deposits accumulated around bone marrow arterioles (Fig. 2c), reminiscent of fatty streaks that evolve into atherosclerotic plaques in other vascular territories. These data document that prototypical vascular pathologies induced by hypertension and atherosclerosis affect the vascular HSPC niche in the bone marrow. Such changed anatomy may modulate leukocyte intravasation and the molecular interactions of endothelial cells with HSPCs that regulate hematopoiesis.

**Fig. 3 |:**
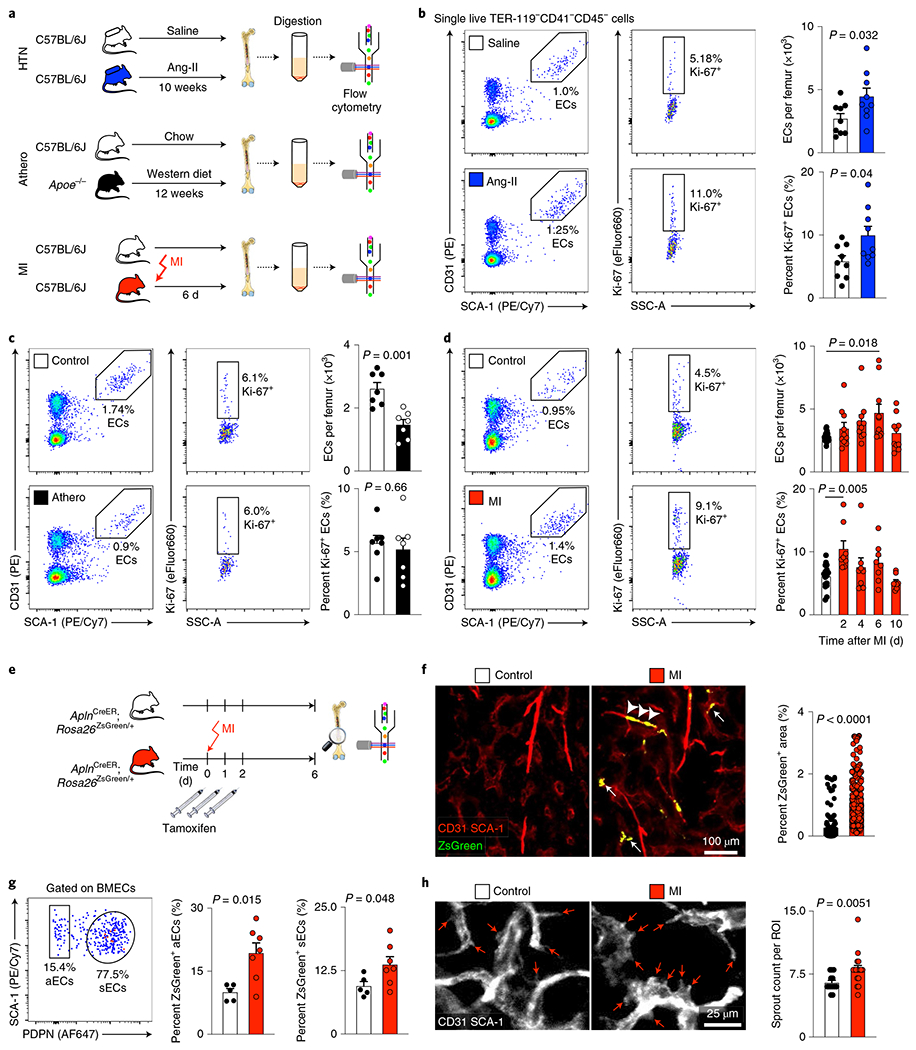
Bone marrow angiogenesis in mice with CVD. **a**, Experimental outline for mouse models of arterial hypertension, atherosclerosis and MI. **b**-**d**, Flow plots and quantification of bone marrow endothelial cells (BMECs) and their proliferation in the femur of mice with hypertension (**b**), atherosclerosis (**c**) and MI (**d**) (*n* = 9 mice per group for hypertension, *n* = 7 for atherosclerosis; two-tailed Welch’s *t*-test; *n* = 20 controls, *n* = 10 for each day after MI for bone marrow endothelial cell (EC) numbers, *n* = 16 controls, *n* = 8 for each day after MI for Ki-67, two-tailed Kruskal-Wallis test with Dunn’s post-test). **e**, Experimental outline. **f**, Immunofluorescence of femurs in *Apln*^CreER^;*Rosa26*^ZsGreen/+^ mice displaying ZsGreen-positive arterioles (arrowheads) and sinusoids (arrows) on day 6 after MI (control, *n* = 214 images of *n* = 10 mice; MI, *n* = 316 images of *n* = 17 mice, two-tailed Mann-Whitney test). **g**, Flow cytometry quantification of new arteriolar (aECs) and sinusoidal endothelial cells (sECs) in the bone marrow of *Apln*^CreER^;*Rosa26*^ZsGreen/+^ mice (*n* = 5 control mice, *n* = 7 with MI, two-tailed Mann-Whitney test). PDPN, podoplanin. **h**, Enumeration of bone marrow blood vessel sprouts (arrows) per region of interest (ROI) in controls and mice 6 d after MI (*n* = 13 ROI from *n* = 6 controls, *n* = 17 ROI from *n* = 5 mice after MI; two-tailed Mann-Whitney test). Data are displayed as mean ± s.e.m.

In mice with hypertension or after MI, arteriolar and sinusoidal vessel density increased in the bone marrow (Fig. 2d,e and Extended Data Fig. 3c,d). No angiogenesis occurred in the skeletal muscle, which served as a control (Extended Data Fig. 3e–g). While bone marrow angiogenesis has been reported in other settings^38,39^, it has not yet been observed in cardiovascular pathologies. In mice with atherosclerosis, the density of bone marrow arterioles, which are thought to harbor quiescent HSPCs^40^, was reduced (Fig. 2e). The angiogenesis phenotype was most prominent in mice with acute MI (Fig. 2e). Therefore, we focused on this acute condition, which rapidly induces robust leukocytosis, in follow-up imaging experiments. Serial intravital microscopy documented increasing marrow vascularity after MI, although not in healthy controls (Fig. 2f–h). Next, we used flow cytometry to enumerate bone marrow endothelial cells and to determine their proliferation rates^13^. Hypertensive mice had elevated bone marrow endothelial cell numbers with higher proliferation rates (Fig. 3a,b), whereas *Apoe*^−/−^ mice with atherosclerosis had fewer bone marrow endothelial cells than age-matched wild-type controls (Fig. 3c). The lower endothelial cell number in bones of mice with atherosclerosis could be caused by insufficient endothelial cell renewal or premature death. In contrast to the bone marrow decline, splenic endothelial cells expanded in mice with atherosclerosis (Extended Data Fig. 3h), a condition that leads to heightened leukocyte production in the spleen^41^.

**Extended Data Fig. 4 |:**
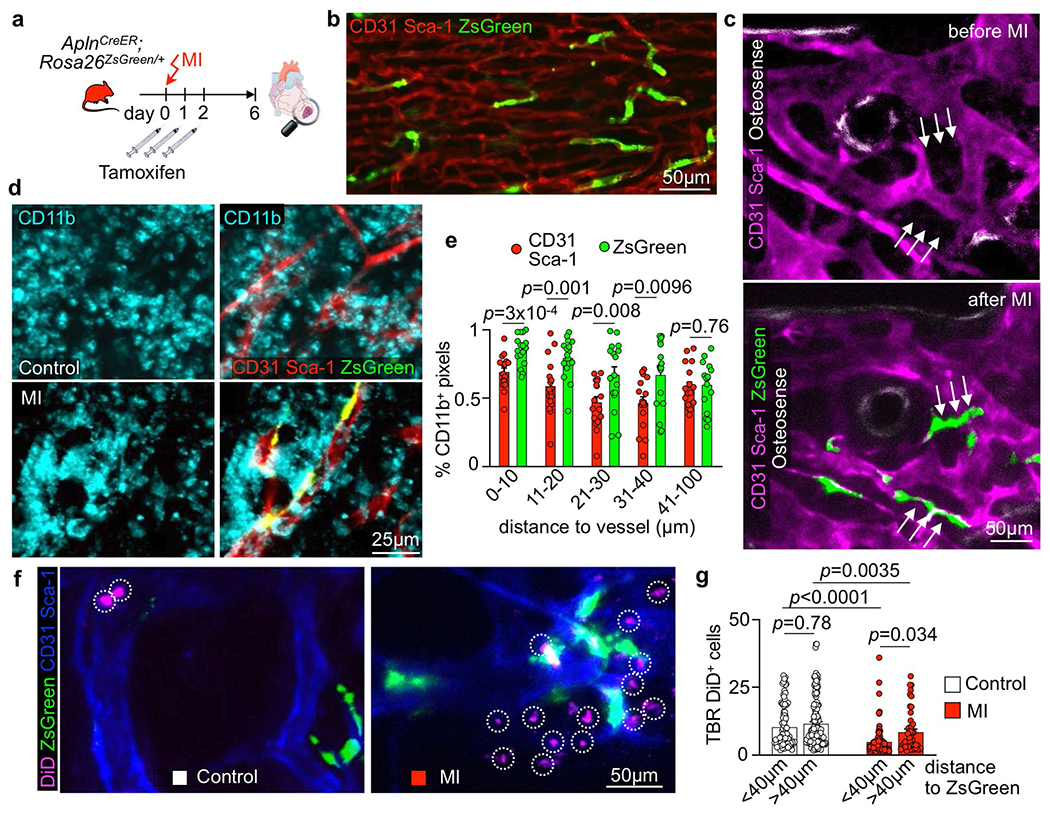
Bone marrow angiogenesis in response to myocardial infarction. **a**, Experimental outline. **b**, Image of the heart’s infarct border zone showing the formation of new ZsGreen^+^ blood vessels after MI (n=3 animals). **c**, Serial intravital immunofluorescence imaging of Tamoxifen-treated *Apln*^*CreER*^*;Rosa26*^*ZsGreen*/+^ mice 1 day before and 6 days after MI, showing newly formed ZsGreen-labelled blood vessels (arrows; n=3 animals per group). **d**, Images of the femur 6 days after MI, showing CD31^+^Sca-1^+^ vasculature, newly formed ZsGreen^+^ blood vessels and CD11b^+^ myeloid cells (n=5 animals). **e**, Distances from leukocyte-containing CD11b^+^ pixels to CD31^+^Sca-1^+^ and to newly formed ZsGreen^+^ vasculature in femora of control and mice after MI (n=17 regions of n=5 animals 6 days post MI, multiple two-tailed t-tests). **f**, In vivo calvarial bone marrow imaging after transplantation of DiD-labelled hematopoietic progenitor cells (LSK, Lin^−^ Sca-1^+^ c-kit^+^) in Tamoxifen-treated *Apln*^*CreER*^*;Rosa26*^*ZsGreen/*+^ mice (naive controls and day 6 after MI). Circles indicate individual DiD-labelled cells (n=3 animals per group). **g**, Mean target-to-background ratios (TBR) of individual DiD-labelled cells at a distance of either <40μm or >40μm to the closest ZsGreen-labelled new blood vessel in controls and mice after MI (control <40μm: n=92 cells analyzed; control >40μm: n=163; MI <40μm: n=98; MI >40μm: n=90; n=3 mice per group; two-way ANOVA with Sidak’s post-test). Data are displayed as mean±SEM.

**Extended Data Fig. 5 |:**
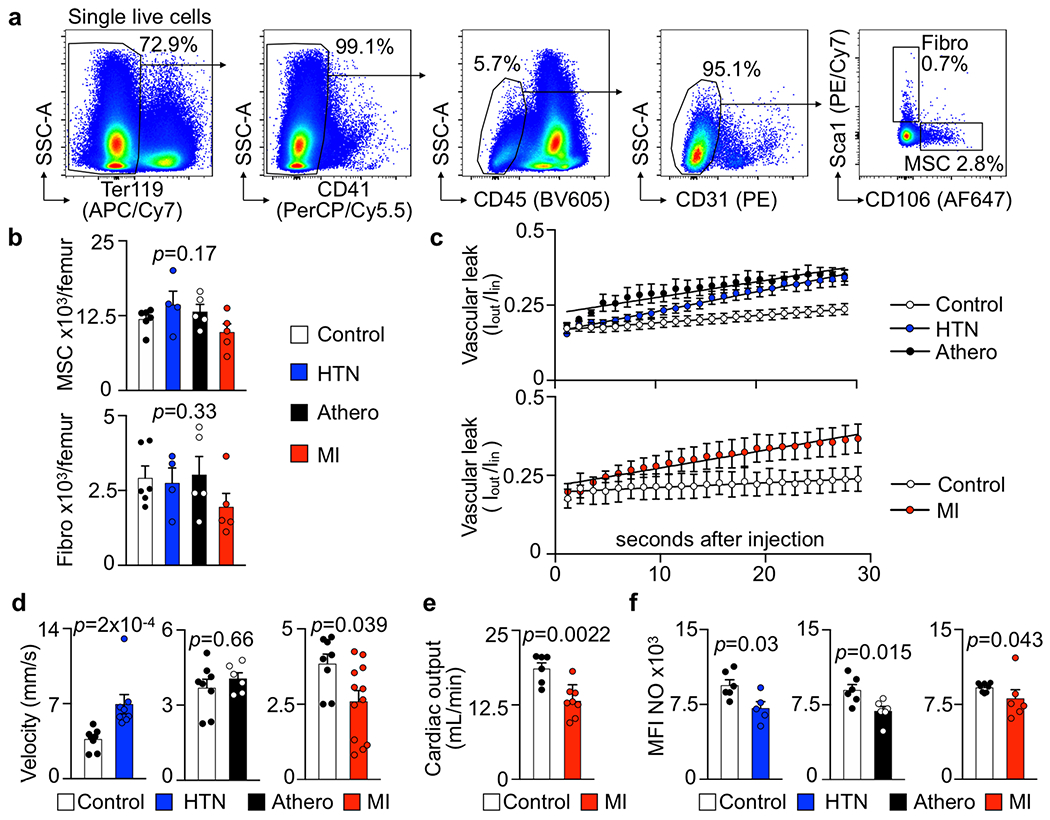
Bone marrow stroma cell composition, vascular leakage, blood flow, and endothelial function in cardiovascular disease. **a**, Flow plots showing the gating strategy for bone marrow mesenchymal stromal cells (MSC) and fibroblasts (Fibro). **b**, Flow cytometry enumeration of bone marrow MSC and fibroblasts (n=6 control mice, n=4 Ang-II induced hypertension (HTN), n=5 *Apoe*^−/−^ with atherosclerosis (Athero) and MI, two-tailed Kruskal-Wallis test). **c**, Quantification of vascular albumin leakage for each time frame acquired by intravital microscopy of the skull bone marrow (n=7 wild type control animals, n=6 Angiotensin II (HTN), n=5 *Apoe*^−/−^ (Athero), n=6 controls for myocardial infarction (MI), n=5 MI). **d**, Mean blood flow velocity in bone marrow arterioles at baseline (n=8 controls, n=8 HTN, n=6 Athero, n=8 controls for MI, n=12 MI, two-tailed Mann-Whitney test). **e**, Cardiac output in controls and mice 3 weeks after MI. Stroke volumes were measured by magnetic resonance imaging (n=6 controls, n=8 MI mice, two-tailed Welch’s *t* test). **f**, Quantification of nitric oxide (NO) in bone marrow endothelial cells by flow cytometry (n=6 saline control, n=5 Ang-II (HTN); n=6 wild type controls, n=6 *Apoe*^−/−^ (Athero); n=8 controls, n=6 MI day 2; two-tailed Mann-Whitney test). Data are displayed as mean±SEM.

Bone marrow endothelial cells were most numerous on day 6 after acute MI with a preceding proliferation peak (Fig. 3d). To verify bone marrow angiogenesis with an orthogonal method, we induced MI in *Apln*^CreER^;*Rosa26*^ZsGreen/+^ reporter mice^42^, in which sprouting endothelial cells and their progeny express ZsGreen (Extended Data Fig. 4a,b). Using confocal imaging on day 6 after MI, we identified newly developed ZsGreen^+^ bone marrow vessels (Fig. 3e,f and Extended Data Fig. 4c). We separately quantified new sinusoidal and arteriolar endothelial cells by flow cytometric staining for the sinusoidal marker podoplanin^13^, noting that ZsGreen^+^ endothelial cell numbers rose after MI for both vascular subtypes (Fig. 3g). New vessels were surrounded by CD11b^+^ myeloid cell clusters (Extended Data Fig. 4d,e) and by transplanted, labeled HSPCs that diluted their membrane dye (Extended Data Fig. 4f,g), a sign of increased progenitor cell division. These data support the notion that post-MI angiogenesis, which was also documented by a higher number of vascular sprouts in the bone marrow (Fig. 3h), and emergency hematopoiesis occur in similar bone marrow regions. None of the tested disease conditions significantly altered mesenchymal stromal cell and fibroblast numbers in the femur (Extended Data Fig. 5a,b). We conclude that CVD profoundly affects structure of bone marrow vasculature and, perhaps most importantly, expands the vascular niche after myocardial ischemia and during hypertension.

### CVD alters vascular function in the bone marrow.

**Fig. 4 |:**
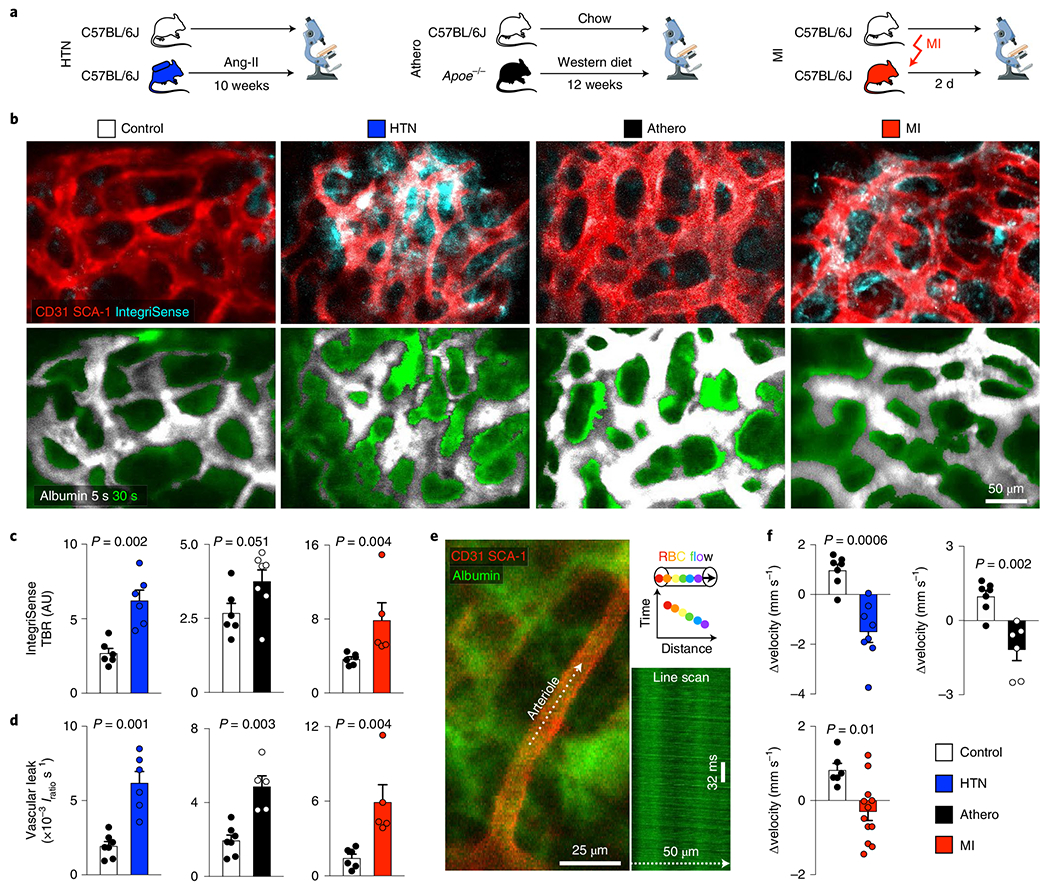
CVD increases integrin abundance, compromises barrier function and leads to endothelial dysfunction in the bone marrow. **a**, Experimental outline. Intravital microscopy images (**b**), quantification of integrin binding (**c**) and albumin extravasation (**d**) in the skull bone marrow (IntegriSense, *n* = 6 control mice for hypertension and atherosclerosis, hypertension (*n* = 6), atherosclerosis (*n* = 7), *n* = 6 controls for MI, MI (*n* = 5); vascular leakage, *n* = 7 controls for hypertension and atherosclerosis, hypertension (*n* = 6), atherosclerosis (*n* = 5), *n* = 6 controls for MI, MI (*n* = 5); two-tailed Mann-Whitney test). AU, arbitrary units; TBR, target-to-background ratio; I_ratio_ = signal intensity outside/inside vessels. Intravital microscopy (**e**) and analysis (**f**) of arteriolar blood flow velocity by line scanning in skull marrow (*n* = 7 controls for hypertension and atherosclerosis, hypertension (*n* = 8), atherosclerosis (*n* = 6); *n* = 6 controls for MI, MI (*n* = 12); two-tailed Mann-Whitney test). RBC, red blood cell. Data are displayed as mean ± s.e.m.

Given how the marrow’s vasculature anatomically adapted to hypertension, atherosclerosis and MI, we reasoned that such changes could lead to altered vascular function. To address this question, we employed imaging to examine functional changes, specifically integrin activation, vascular leakage and blood flow in the three examined conditions. Intravital microscopy indicated increased binding of a fluorescent RGD-containing probe with affinity for integrin αVβ3 (refs. ^43,44^) in the skull of mice with hypertension or MI (Fig. 4a–c). This result may reflect integrin activation during the angiogenic response^45^, which we observed in mice with MI or hypertension.

Concomitantly, we studied endothelial barrier function with intravital time-lapse imaging after labeling the blood pool with fluorescent albumin^44^. In all three conditions, vascular leakage accelerated (Fig. 4b,d and Extended Data Fig. 5c). This impairment in barrier function, in conjunction with the observed integrin activation, may facilitate trafficking of cells and blood-borne signals across the bone marrow endothelium, potentially supporting HSPC activation by circulating alarmins and reactive oxygen species, processes that augment leukocytosis.

One of the primary functions of the vasculature is to facilitate and regulate blood flow. We therefore used intravital microscopy to assay blood velocity in the skull bone marrow (Fig. 4e), which increased in bone marrow arterioles of hypertensive mice (Extended Data Fig. 5d). These data agree with the notion that, during hypertension, fibrosis (Extended Data Fig. 3b) and a higher myogenic tone systemically reduce the distensibility of vessels^46^. Blood flow remained unchanged in *Apoe*^−/−^ mice with atherosclerosis and declined after MI (Extended Data Fig. 5d). We attribute the lower blood flow velocity after MI to a decrease in cardiac output (Extended Data Fig. 5e). To probe for the CVD hallmark endothelial dysfunction^47^, that is, the reduced ability of arterial resistance blood vessels to dilate in response to acetylcholine, we next quantified blood velocity in skull marrow microvessels after intravenous (i.v.) injection of acetylcholine. Acetylcholine elicits nitric oxide release from the healthy endothelium, which induces arterial vasodilation and increases flow in downstream vascular beds^48,49^. As expected for healthy vasculature, acetylcholine increased velocity in bone marrow arterioles of control mice. In contrast, velocity decreased after acetylcholine injection in mice with hypertension, atherosclerosis or MI (Fig. 4e,f). This impaired vasodilation response in the bone marrow of mice with CVD is typical for endothelial vasomotor dysfunction. Using flow cytometry of bone marrow endothelial cells collected from hypertensive, atherosclerotic and post-MI mice, we indeed found reduced levels of endothelial nitric oxide (Extended Data Fig. 5f). The altered hemodynamics in the bone marrow of mice with CVD also raise the question whether mechanosensing, which influences lymphopoiesis^50^, participates in regulating myelopoiesis during CVD.

**Fig. 5 |:**
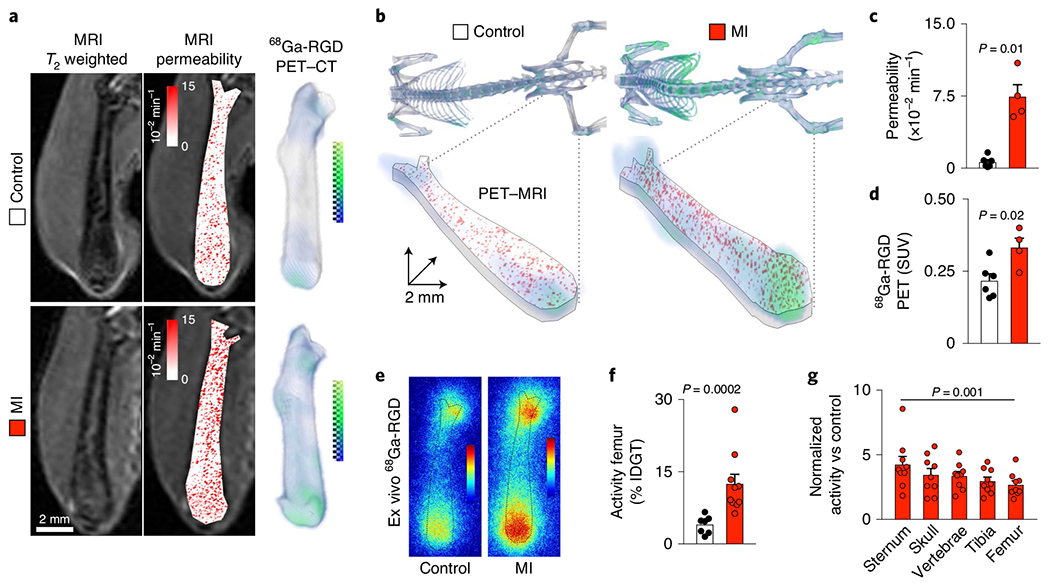
MI leads to integrin activation and vascular permeability in the bone marrow. **a**, In vivo precontrast *T_2_*-weighted rapid acquisition with refocused echoes (RARE) MRI (left), post-contrast parametric map of the permeability surface area product (MRI permeability, middle) and three-dimensional (3D) PET-computed tomography (CT) of ^68^Ga-RGD uptake in the femur (right). **b**, ^68^Ga-RGD PET-CT fused with MRI parametric permeability maps. In vivo mean permeability (**c**) and PET standardized uptake value (SUV) (**d**) (*n* = 6 control mice, *n* = 4 with MI, two-tailed Mann-Whitney test). **e**, ^68^Ga-RGD autoradiography of femora. **f**, Scintillation counting of femora yielding percent injection dose per g tissue (IDGT) (*n* = 7 control mice, *n* = 10 for MI, two-tailed Mann-Whitney test). **g**, Post-MI ^68^Ga-RGD uptake in different bone marrow locations normalized to that in the control (*n* = 9 mice for all, two-sided Friedman test with Dunn’s post-test). Data are displayed as mean ± s.e.m.

Next, we complemented our intravital microscopy studies with non-invasive whole-mouse imaging, enabling system-wide in vivo assessment of all hematopoietic sites. To this end, an RGD-containing gallium-68 positron emission tomography (PET) reporter (^68^Ga-RGD) and a gadolinium-labeled albumin derivative were co-injected into mice with MI, followed by a previously validated PET–magnetic resonance imaging (MRI) protocol^44^. This multimodal dataset, which relied on imaging agents corresponding to the fluorescent companions used for intravital microscopy, indicated increased integrin binding and higher vascular permeability in the femur after MI (Fig. 5a–d). Autoradiography (Fig. 5e) and scintillation counting (Fig. 5f) confirmed greater ^68^Ga-RGD accumulation in the bones of mice with acute MI. Analysis of ^68^Ga-RGD in various hematopoietic sites identified the highest activity in the sternum (Fig. 5g), possibly due to its proximity to the infarcted heart. Together, these cellular and whole-mouse imaging data indicate that hypertension, atherosclerosis and MI induce not only anatomical but also functional vascular changes in the bone marrow.

### Blocking VEGFR2 curbs bone marrow angiogenesis and emergency hematopoiesis after MI.

**Extended Data Fig. 6 |:**
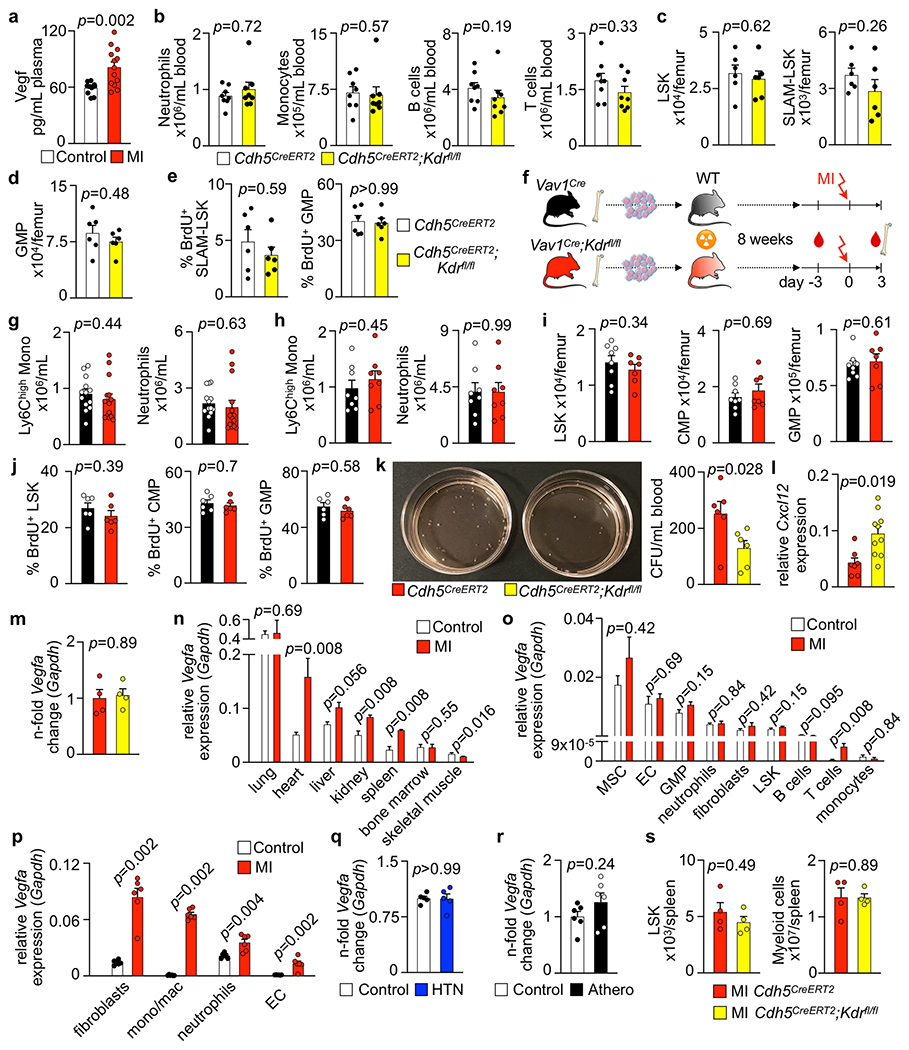
Endothelial Vegf receptor 2 (Vegfr2) signaling in myocardial infarction. **a**, Vegf in blood plasma of controls and 3 days post myocardial infarction (MI) by ELISA (control n=12, MI n=13, two-tailed Welch’s *t*-test). **b-e**, Blood leukocyte (**b**), bone marrow HSPC numbers (**c, d**), and proliferation (**e**) obtained in naive *Cdh5*^*CreERT2*^ and *Cdh5*^*CreERT2*^*;Kdr*^*fl/fl*^ mice. Both groups were treated with Tamoxifen (B, n=8 mice per group; C-E, n=6 mice per group; two-tailed Mann-Whitney test). **f**, Experimental outline. Bone marrow mononuclear cells (BMNC) were isolated from *Vav1*^*Cre*^ and *Vav1*^*Cre*^*;Kdr*^*f/fl*^ mice and transplanted into lethally irradiated wild type recipients. 8 weeks after bone marrow transplantation (BMT), baseline blood data were obtained by flow cytometry. Both groups were then subjected to MI.**g**, Blood monocyte and neutrophil numbers 8 weeks post BMT (n=13 *Vav1*^*Cre*^ recipients, n=15 *Vav1*^*Cre*^*;Kdr*^*f/fl*^ recipients, two-tailed Welch’s *t*-test). **h**, Blood myeloid cell numbers 3 days post MI (n=8 mice per group, two-tailed Welch’s t-test). **i, j**, Bone marrow Lin^−^ Sca-1^+^ c-kit^+^ (LSK), common myeloid progenitor (CMP) and granulocyte/monocyte progenitor (GMP) numbers (**i**) (n=8 *Vav1*^*Cre*^ recipients, n=7 *Vav1*^*Cre*^*;Kdr*^*fl/fl*^ recipients, two-tailed Mann-Whitney test) and proliferation (**j**) 3 days post MI (n=6 mice per group, two-tailed Mann-Whitney test). **k**, Image and analysis of colony forming units (CFU) for hematopoietic progenitor cells in blood taken 3 days after MI (n=6 mice pre group, two-tailed Mann-Whitney test). **l**, *Cxcl12* expression in flow-sorted bone marrow endothelial cells on day 2 after MI (n=7 *Cdh5*^*CreERT2*^, n=10 *Cdh5*^*CreERT2*^*;Kdr*^*f/fl*^, two-tailed Mann-Whitney test). **m**, Relative expression of *Vegfa* in flow sorted BMEC isolated from *Cdh5*^*CreERT2*^ and *Cdh5*^*CreERT2*^*;Kdr*^*f/fl*^ mice 3 days post MI by qPCR (n=4 mice per group, two-tailed Mann-Whitney test). **n**, *Vegfa* expression in different organs in controls and 48hrs post MI (n=5 mice per group, individual two-tailed Mann-Whitney tests comparing control and MI for each organ). **o**, mRNA levels of *Vegfa* (in relation to *Gapdh*) in FACS-isolated bone marrow cell populations from controls and mice 48hrs after MI (n=5 mice per group, individual two-tailed Mann-Whitney tests comparing control and MI for each cell type). **p**, *Vegfa* gene expression on day 2 after myocardial infarction. Relative gene expression of *Vegfa* in cell populations flow-sorted from leftventricular healthy (Control) and infarct tissue (MI) in relation to *Gapdh*, as measured by qPCR (n=6 mice per group, two-sided Mann-Whitney test for all).**q**, Relative *Vegfa* mRNA assessed by qPCR in bone marrow endothelial cells (BMEC) FACS-isolated from mice with saline or Angiotensin II (HTN) minipumps for 8 weeks (n=5 mice per group, two-tailed Mann-Whitney test). **r**, *Vegfa* expression in BMEC from wild type (Control) and *Apoe*^−/−^ mice on a Western Diet for 12 weeks (Athero) (n=6 mice per group, two-tailed Mann-Whitney test). **s**, Flow cytometry of splenic LSK and myeloid cells in *Cdh5*^*CreERT2*^ controls and *Cdh5*^*CreERT2*^*;Kdr*^*f/fl*^ mice 3 days post MI (n=4 mice per group, two-tailed Mann-Whitney test). Data are displayed as mean±SEM.

**Fig. 6 |:**
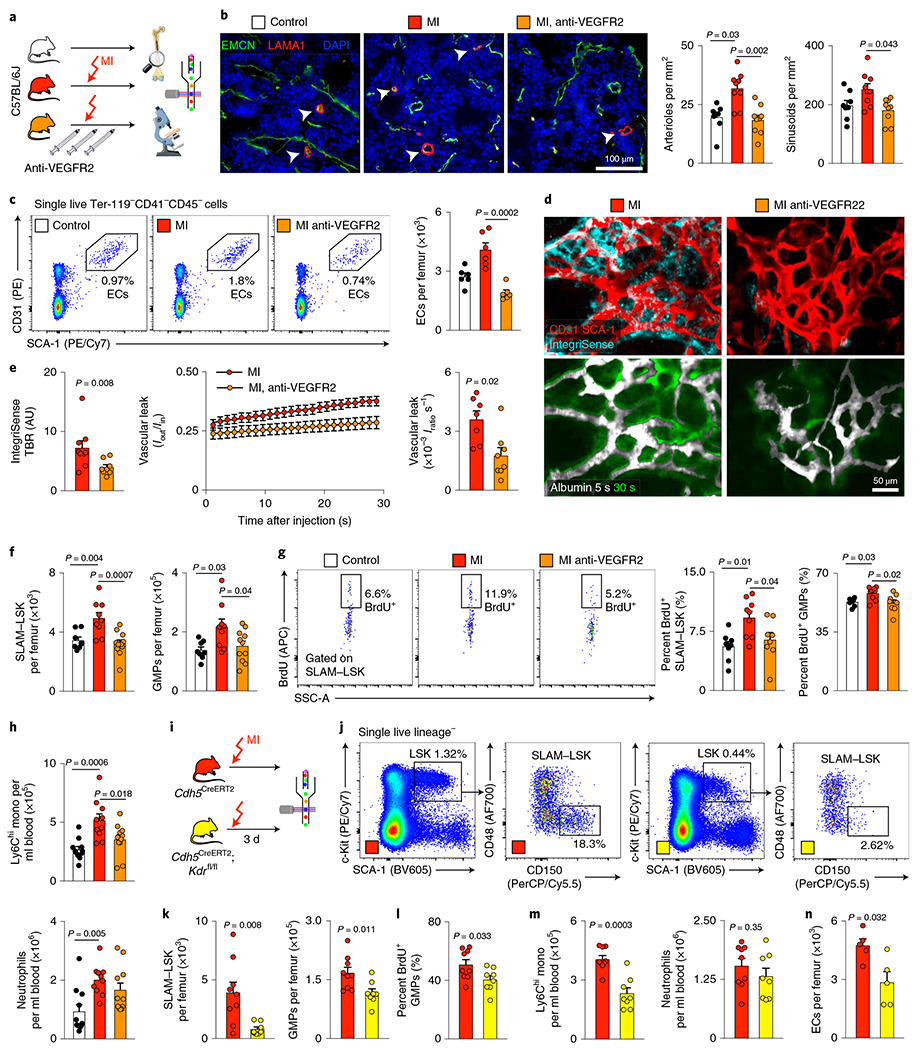
VEGF signaling expands angiogenesis and enables emergency hematopoiesis after MI. **a**, Experimental outline. **b**, Immunofluorescence of EMCN-positive sinusoids and LAMA1^+^ arterioles (arrowheads) in the femur of controls, 6 d after MI and 6 d after MI with anti-VEGFR2 treatment (*n* = 8 controls, *n* = 9 with MI, *n* = 9 with MI and anti-VEGFR2; two-tailed Kruskal-Wallis test with Dunn’s post-test). **c**, Flow plots and bone marrow endothelial cell numbers in the femur (*n* = 7 controls, *n* = 6 with MI, *n* = 8 with MI and anti-VEGFR2 treatment; two-tailed Kruskal-Wallis test with Dunn’s post-test). Intravital microscopy (**d**) and analysis of integrin binding and vascular permeability (**e**) in skull bone marrow 2 d after MI (IntegriSense, MI (*n* = 9 mice), MI with anti-VEGFR2 treatment (*n* = 8); vascular leakage, MI (*n* = 7), MI with anti-VEGFR2 treatment (*n* = 8); two-tailed Mann-Whitney test). Frequency (**f**) and proliferation (**g**) of SLAM-LSK and GMPs in the femur (numbers, *n* = 8 controls, *n* = 9 with MI (day 3), *n* = 11 with MI and anti-VEGFR2 treatment; proliferation, *n* = 8 controls, *n* = 9 with MI (day 3), *n* = 8 with MI and anti-VEGFR2 treatment; one-way ANOVA with Holm-Sidak’s post-test). BrdU, bromodeoxyuridine. **h**, Blood monocyte (mono) and neutrophil numbers by flow cytometry (*n* = 11 controls, *n* = 10 with MI (day 3), *n* = 11 with MI and anti-VEGFR2 treatment; one-way ANOVA with Holm-Sidak’s post-test). **i**, Experimental outline. Flow plots (**j**), numbers (**k**) and proliferation (**l**) of HSPCs in the femora of *Cdh5*^CreERT2^ controls and *Cdh5*^CreERT2^;*Kdr*^fl/fl^ mice 3 d after MI (*n* = 9 *Cdh5*^CreERT2^ mice, *n* = 8 *Cdh5*^CreERT2^;*Kdr*^fl/fl^ mice; two-tailed Welch’s *t*-test). Both groups received tamoxifen injections 2 weeks before MI surgery. **m**, Blood monocytes and neutrophils 3 d after MI (*n* = 9 *Cdh5*^CreERT2^ mice, *n* = 8 *Cdh5*^CreERT2^;*Kdr*^fl/fl^ mice; two-tailed Welch’s *t*-test). **n**, Bone marrow endothelial cells per femur on day 7 after MI (*n* = 5 mice per group, two-tailed Mann-Whitney test). Data are displayed as mean ± s.e.m.

Among the vascular changes induced by CVD, post-MI angiogenesis appeared the most profound. Interestingly, this robust angiogenic phenotype occurred in synchrony with the timeline of emergency hematopoiesis^32^ and leukocytosis^27^ induced during MI. As endothelial cells regulate HSPC proliferation^12^, we wondered whether the observed expansion of the vascular bone marrow niche influences post-MI leukocytosis. Specifically, we hypothesized that post-MI angiogenesis in the bone marrow facilitates emergency hematopoiesis, perhaps by expanding the vascular niche in which myelopoiesis occurs. Motivated by a prior report that VEGFR2 is essential for vascular recovery after irradiation^38^, we evaluated VEGF signaling after MI. VEGF protein levels were indeed higher in the blood of mice with MI (Extended Data Fig. 6a), a result that matches published data obtained in patients^51^. Inhibiting the cognate receptor VEGFR2 with a neutralizing antibody abolished the post-MI increase in bone marrow arterioles, sinusoids and endothelial cells (Fig. 6a–c). In line with these ex vivo data, intravital microscopy detected reduced presence of integrins and less vascular leakage, which increase during angiogenesis^45^, in post-MI mice treated with VEGFR2-blocking antibody (Fig. 6d,e and Supplementary Videos 1–3). More importantly, VEGFR2 inhibition also promoted HSPC quiescence (Fig. 6f,g) and lowered the amount of circulating Ly6C^hi^ monocytes (Fig. 6h) after MI. We reasoned that reduced myelopoiesis could either be secondary to inhibiting bone marrow angiogenesis or alternatively could be caused by blocking VEGFR2 on hematopoietic cells^52^. To distinguish between these possibilities, we bred *Cdh5*^CreERT2^ mice with *Kdr*^fl/fl^ mice for tamoxifen-inducible, endothelial cell-specific deletion of *Vegfr2.* In these mice, in which baseline hematopoiesis was unaffected (Extended Data Fig. 6b–e), post-MI hematopoiesis and numbers of circulating Ly6C^hi^ monocytes (Fig. 6i–m) declined, indicating that VEGF signaling affects leukocytosis via bone marrow endothelial cells. *Vegfr2* deletion from hematopoietic cells of *Vav1*^Cre^;*Kdr*^fl/fl^ mice, the bone marrow of which was transplanted into wild-type recipients, did not affect steady-state myeloid blood numbers (Extended Data Fig. 6f,g) or post-MI hematopoiesis (Extended Data Fig. 6h–j), ruling out that hematopoietic cell sensing of VEGF expands post-MI leukocyte production.

Comparable to mice treated with anti-VEGFR2 therapy, *Cdh5*^CreERT2^;*Kdr*^fl/fl^ animals, in which endothelial cells lack expression of *Vegrf2*, showed no endothelial cell expansion in response to MI (Fig. 6n), along with diminished HSPC release from the bone marrow (Extended Data Fig. 6k). In bone marrow endothelial cells sorted by flow cytometry, *Cxcl12*, encoding a niche factor required for HSPC maintenance^12^ and retention^53,54^, was expressed at higher levels in *Cdh5*^CreERT2^;*Kdr*^fl/fl^ mice than in *Cdh5*^CreERT2^ littermate controls, both with MI (Extended Data Fig. 6l). Endothelial cell *Vegfa* expression was unaffected in these mice (Extended Data Fig. 6m).

Evaluating potential sources of the receptor ligand VEGFA, we found that its expression after MI rose in the heart, kidney and spleen but not in whole bone marrow or bone marrow endothelial cells (Extended Data Fig. 6n,o). These data indicate that the increased VEGFA plasma levels in patients and mice with MI originate outside the bone marrow, perhaps in ischemic tissue with decreased oxygen tension^55^. Indeed, 2 d after MI, *Vegfa* expression rose in both stromal and myeloid infarct cells (Extended Data Fig. 6p). *Vegfa* expression by bone marrow endothelial cells was also unchanged in mice with hypertension or atherosclerosis (Extended Data Fig. 6q,r), and post-MI hematopoiesis in the spleen was not significantly affected in *Cdh5*^CreERT2^;*Kdr*^fl/fl^ mice (Extended Data Fig. 6s). Together, these data show that post-MI emergency hematopoiesis is licensed by the expanding bone marrow vasculature and requires VEGFR2 signaling in bone marrow endothelial cells.

### Bone marrow endothelium is activated in hypertension, atherosclerosis and MI.

**Fig. 7 |:**
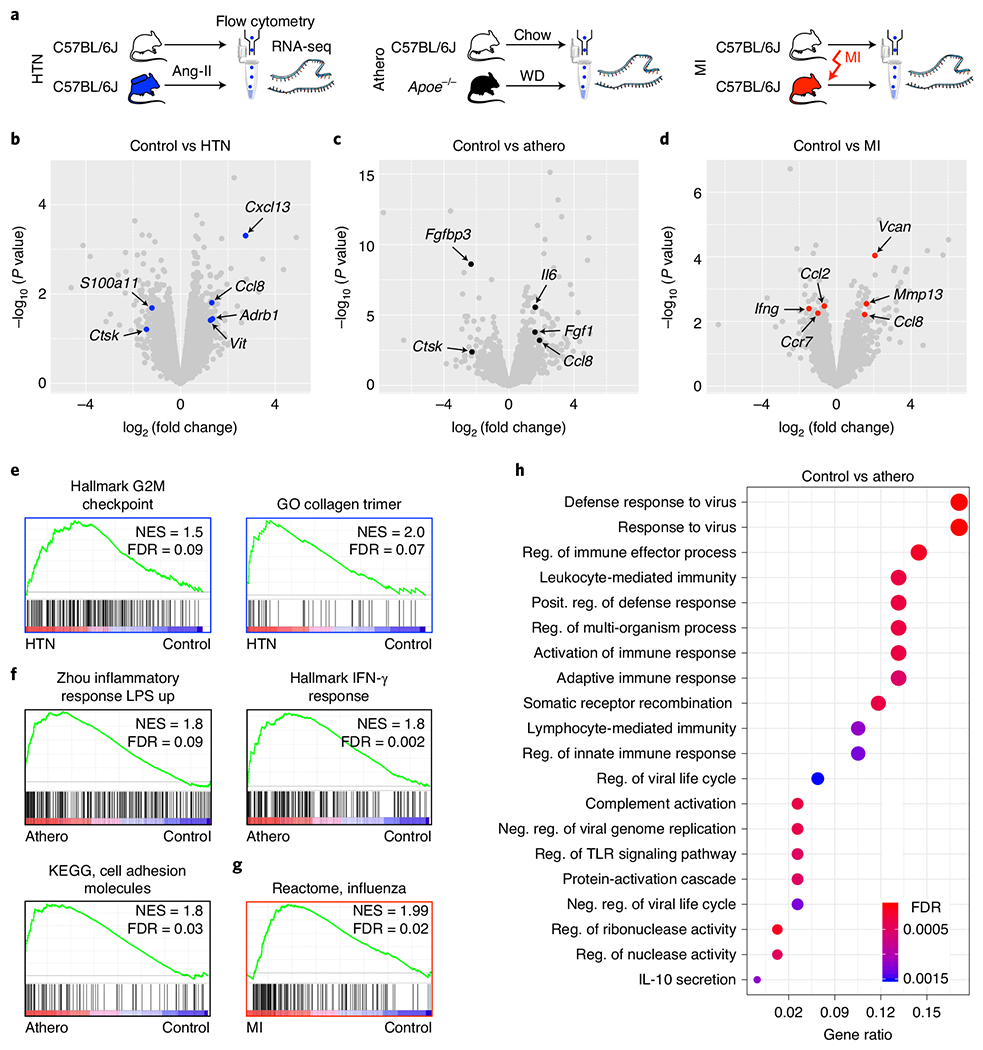
CVD induces inflammatory bone marrow endothelial cell gene expression. **a**, Experimental outline. WD, western diet. **b**–**d**, Differential gene expression in bone marrow endothelial cells assessed by RNA-seq (*n* = 3 mice per group). **e**–**g**, GSEA plots of gene sets related to inflammation. NES, normalized enrichment score; FDR, false discovery rate; GO, gene ontology; IFN-γ; interferon-γ; KEGG, Kyoto Encyclopedia of Genes and Genomes; LPS, lipopolysaccharide. **h**, Plot of gene set enrichment in *Apoe*^−/−^ mice compared to controls. Neg., negative; posit., positive; reg., regulation; TLR, Toll-like receptor.

Thus far, our data document profound anatomic and functional vascular adaptions to CVD, with relevance for leukocytosis. To explore how CVD affects the transcriptional program of bone marrow endothelial cells, that is, the signaling that contributes to the observed phenotypic adaptions in the bone marrow, we used RNA-seq to compare bone marrow endothelial cells isolated by flow cytometry from naive controls to cells collected from hypertensive mice, to *Apoe*^−/−^ mice with atherosclerosis and to mice 4 d after MI (Supplementary Table 1 and Fig. 7a–h). Hypertension led to enrichment of gene sets related to cell proliferation and fibrosis (Fig. 7e), matching our observation of angiogenesis and vascular fibrosis in the bone marrow in this condition. Gene set enrichment analysis (GSEA) revealed inflammatory endothelial cell activation in mice with atherosclerosis and MI (Fig. 7f,g). In atherosclerosis, we observed enrichment of genes related to adhesion molecules, interferon and Toll-like receptor signaling (Fig. 7f,h). These findings, which agree with the observed anatomic and functional changes described above, align with our overarching hypothesis that the inflammatory endothelial response to CVD^56–59^ extends to the bone marrow’s vascular niche.

To explore whether CVD-induced expression changes in bone marrow endothelial cells influence the function of the vascular niche, its interaction with HSPCs and consequently leukocytosis, we next deleted top-ranked genes identified by RNA-seq, one each in *Apoe*^−/−^ mice with atherosclerosis and mice with acute MI. We selected atherosclerosis and MI because in these conditions, the disease-promoting properties of leukocytes are documented extensively^5,19–22,25,27,28^.

### IL-6 synthesized by endothelial cells boosts myelopoiesis and leukocytosis in atherosclerosis.

**Extended Data Fig. 7 |:**
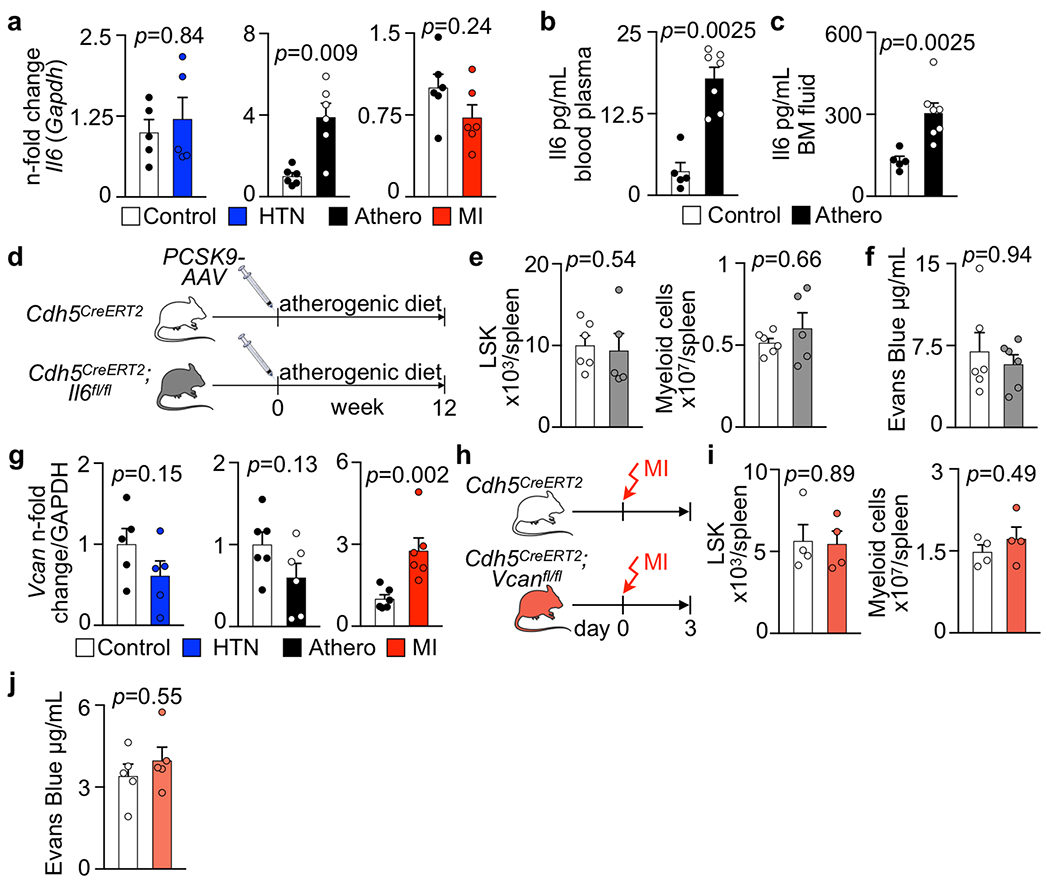
Gene deletion in bone marrow endothelial cells. **a**, Relative *Il6* mRNA levels assessed by qPCR in bone marrow endothelial cells (BMEC) FACS-isolated from mice with saline or Angiotensin II (HTN) for 8 weeks (n=5 mice per group), from wild type (Control) and *Apoe*^−/−^ mice on a Western Diet for 10 weeks (Athero, n=6 mice per group), and from controls and mice on day 4 after MI (n=6 mice per group, two-tailed Mann-Whitney test). **b, c**, Concentration of Il6 in blood plasma (**b**) and bone marrow interstitial fluid (**c**) in wild type (Control) and *Apoe*^−/−^ mice on a Western Diet for 10 weeks (Athero) by ELISA (n=5 controls, n=7 *Apoe*^−/−^, two-tailed Mann-Whitney test). **d**, Experimental outline. **e**, Flow cytometry enumeration of splenic LSK and myeloid cells in *Cdh5*^*CreERT2*^ and *Cdh5*^*CreERT2*^*;Il6*^*fl/fl*^ mice 12 weeks after *PCSK9-AAV* injection and start of an atherogenic diet in order to induce atherosclerosis (n=6 *Cdh5*^*CreERT2*^, n=5 *Cdh5*^*CreEBT2*^*;Il6*^*f/fl*^ mice, two-tailed Mann-Whitney test). **f**, Vascular permeability measured by Evans Blue extravasation in the femoral bone marrow of *Cdh5*^*CreERT2*^ and *Cdh5*^*CreERT2*^*;Il6*^*fl/fl*^ mice with atherosclerosis (n=6 per group, two-tailed Mann-Whitney test). **g**, *Versican (Vcan)* expression in flow-sorted bone marrow endothelial cells from mice with saline or Angiotensin II (HTN) for 8 weeks (n=5 mice per group), from wild type (Control) and *Apoe*^−/−^ mice on a Western Diet for 10 weeks (n=6 mice per group) and from controls and mice 4 days after MI (n=6 mice per group, two-tailed Mann-Whitney tests). **h**, Experimental outline. **i**, Flow cytometry enumeration of splenic LSK and myeloid cells in *Cdh5*^*CreERT2*^ and *Cdh5*^*CreERT2*^*;Vcan*^*fl/fl*^ mice 3 days after MI (n=4 mice per group, two-tailed Mann-Whitney test). **j**, Vascular permeability measured by Evans Blue extravasation in the femoral bone marrow of *Cdh5*^*CreERT2*^ and *Cdh5*^*CreERT2*^*;Vcan*^*fl/fl*^ mice on day 2 after MI (n=5 mice per group, two-tailed Mann-Whitney test). Data are displayed as mean±SEM.

**Fig. 8 |:**
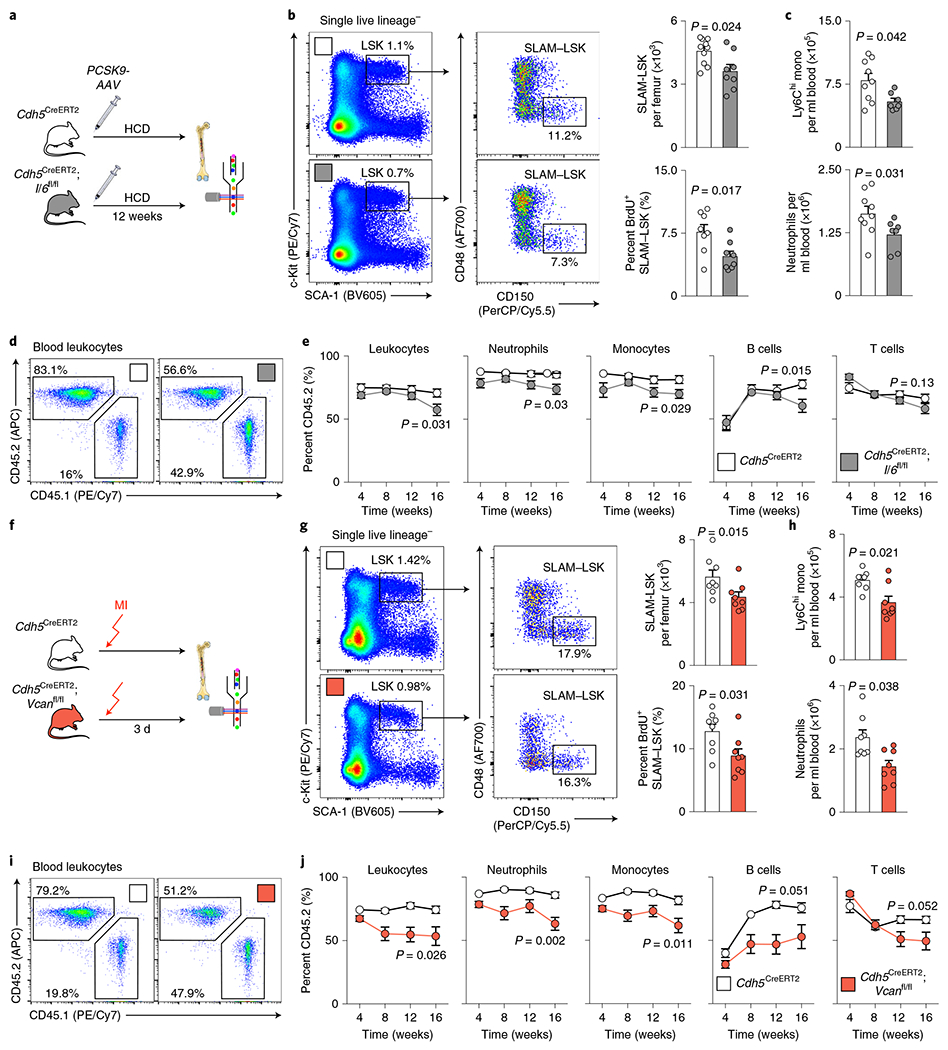
IL-6 and versican derived from bone marrow endothelial cells boost leukocytosis in atherosclerosis and MI. **a**, Experimental outline. Atherosclerosis was induced in *Cdh5*^CreERT2^ and *Cdh5*^CreERT2^;*Il6*^fl/fl^ mice by injecting *PCSK9-AAV* and administering a high-cholesterol diet (HCD) for 12 weeks. Both groups received tamoxifen injections 2 weeks before AAV injection and the start of the high-cholesterol diet. **b**, Flow plots, frequency and proliferation of SLAM-LSK in *Cdh5*^CreERT2^ and *Cdh5*^CreERT2^;*Il6*^fl/fl^ mice with atherosclerosis (*n* = 10 *Cdh5*^CreERT2^ mice, *n* = 8 *Cdh5*^CreERT2^;*Il6*^fl/fl^ mice for numbers; *n* = 8 mice per group for BrdU; two-tailed Welch’s *t*-test). **c**, Blood monocyte and neutrophil numbers (*n* = 9 *Cdh5*^CreERT2^ mice, *n* = 7 *Cdh5*^CreERT2^;*Il6*^fl/fl^ mice; two-tailed Mann-Whitney test). **d**,**e**, Competitive bone marrow-transplantation assay. Bone marrow mononuclear cells (BMNCs) were isolated from *Cdh5*^CreERT2^ and *Cdh5*^CreERT2^;*Il6*^fl/fl^ mice with atherosclerosis and mixed with BMNCs from naive CD45.1 mice in equal proportions before transplantation into lethally irradiated CD45.2 recipients. Flow plot (**d**) and time course (**e**) of blood leukocyte chimerism, showing reduced contribution from *Cdh5*^CreERT2^;*Il6*^fl/fl^ donors (*n* = 8 recipient mice per group, two-tailed Welch’s *t*-test for percent CD45.2 at week 16). **f**, Experimental outline. *Cdh5*^CreERT2^ and *Cdh5*^CreERT2^;*Vcan*^fl/fl^ mice received tamoxifen injections 2 weeks before MI. **g**,**h**, Flow plots, numbers and proliferation of bone marrow SLAM-LSK (**g**) and blood myeloid cells (**h**) in *Cdh5*^CreERT2^ controls and *Cdh5*^CreERT2^;*Vcan*^fl/fl^ mice, all 3 d after MI (*n* = 8 mice per group, two-tailed Mann-Whitney test). **i**,**j**, Competitive bone marrow-transplantation assay. BMNCs were taken from *Cdh5*^CreERT2^ and *Cdh5*^CreERT2^;*Vcan*^fl/fl^ mice, both 3 d after MI, and mixed with BMNCs from naive CD45.1 mice in equal proportions before transplantation into lethally irradiated CD45.2 recipients. Flow plot (**i**) and time course (**j**) of blood leukocyte chimerism, showing reduced contribution from *Cdh5*^CreERT2^;*Vcan*^fl/fl^ donors (*n* = 8 recipient mice per group, two-tailed Welch’s *t*-test for percent CD45.2 at week 16). Data are displayed as mean ± s.e.m.

Interleukin (IL)-6, a pro-inflammatory cytokine for which levels increase systemically in atherosclerosis^60,61^ and that endothelial cells express in response to inflammatory mediators^62,63^, was chosen for follow-up because its transcript was highly ranked by *P* value among the upregulated transcripts in atherosclerosis (Fig. 7c and Supplementary Table 1). While *Il6* expression was unchanged in hypertension or MI, higher *Il6* expression was confirmed by real-time PCR in bone marrow endothelial cells from *Apoe*^−/−^ mice (Extended Data Fig. 7a). IL-6 protein levels increased in the blood and bone marrow of mice with atherosclerosis (Extended Data Fig. 7b,c). Further support for choosing *Il6* for a loss-of-function study consisted of the following: (1) HSPCs express the cognate receptor for this cytokine (https://www.immgen.org); (2) the risk of MI increases 2.3-fold in patients with IL-6 levels in the highest quartile^64^; and (3) a single-nucleotide polymorphism (p.Asp358Ala) in the *IL6R* gene is linked to a reduced odds ratio for coronary heart disease^65^. To test whether the increase in endothelial IL-6 production during atherosclerosis contributes to the leukocytosis that fuels arterial inflammation^19–22,25^, we induced atherosclerosis in *Cdh5*^CreERT2^;*Il6*^fl/fl^ mice using an atherogenic diet and adeno-associated virus (AAV)-mediated gene transfer of mutant human *PCSK9.* Compared to *Cdh5*^CreERT2^ littermate controls, endothelial cell-specific *Il6* deletion reduced HSPC numbers and proliferation in the bone marrow and lowered systemic myeloid cell numbers (Fig. 8a–c), while splenic hematopoiesis remained unchanged (Extended Data Fig. 7d,e). A competitive bone marrow-transplantation assay comparing *Cdh5*^CreERT2^;*Il6*^fl/fl^ to *Cdh5*^CreERT2^ bone marrow obtained from mice with atherosclerosis revealed a lower blood leukocyte chimerism for cells originating from donors with endothelial deletion of *Il6* (Fig. 8d,e). In *Cdh5*^CreERT2^;*Il6*^fl/fl^ mice, vascular leakage in the bone marrow was unaffected (Extended Data Fig. 7f). In sum, these data indicate that, in mice with atherosclerosis, endothelial IL-6 production augments HSPC proliferation and leukocytosis and, more generally, that endothelial cell interactions with HSPCs are pathologically altered in atherosclerosis. While hyperlipidemia is a likely instigator of increased IL-6 expression by bone marrow endothelial cells^63^, we cannot formally rule out that vascular beds outside the bone marrow contributed to the observed phenotypes.

### Endothelial cell-derived versican augments myelopoiesis and leukocytosis after MI.

*Vcan* (encoding versican) was chosen for a functional follow-up study because it ranked highly among the genes expressed at higher levels in bone marrow endothelial cells after MI (Fig. 7d and Supplementary Table 1). The gene encodes a proteoglycan of the extracellular matrix that localizes in the arterial intima and adventitia. Increased *Vcan* expression after MI was confirmed by real-time PCR in bone marrow endothelial cells isolated by flow cytometry but was unchanged in bone marrow endothelial cells from mice with hypertension or atherosclerosis (Extended Data Fig. 7g). Generating functional networks with hyaluronan^66^, versican regulates cell adhesion, proliferation and angiogenesis^67^, processes that we observed in the marrow after MI (Fig. 2d and following). Therefore, we deleted *Vcan* in endothelial cells to test whether versican affects hematopoiesis. In *Cdh5*^CreERT2^;*Vcan*^fl/fl^ mice, we detected reduced emergency hematopoiesis after MI in the bone marrow (Fig. 8f,g). The lower hematopoietic response dampened post-MI leukocytosis in *Cdh5*^CreERT2^;*Vcan*^fl/fl^ mice (Fig. 8h). A competitive bone marrow-transplantation assay yielded a lower leukocyte chimerism for cells derived from *Cdh5*^CreERT2^;*Vcan*^fl/fl^ mice with MI (Fig. 8i,j), confirming that endothelial cell-specific *Vcan* deletion reduced the abundance of upstream HSCs after MI. *Vcan* deletion did not affect splenic hematopoiesis or the bone marrow’s vascular permeability after MI (Extended Data Fig. 7h–j).

## Discussion

**Extended Data Fig. 8 |:**
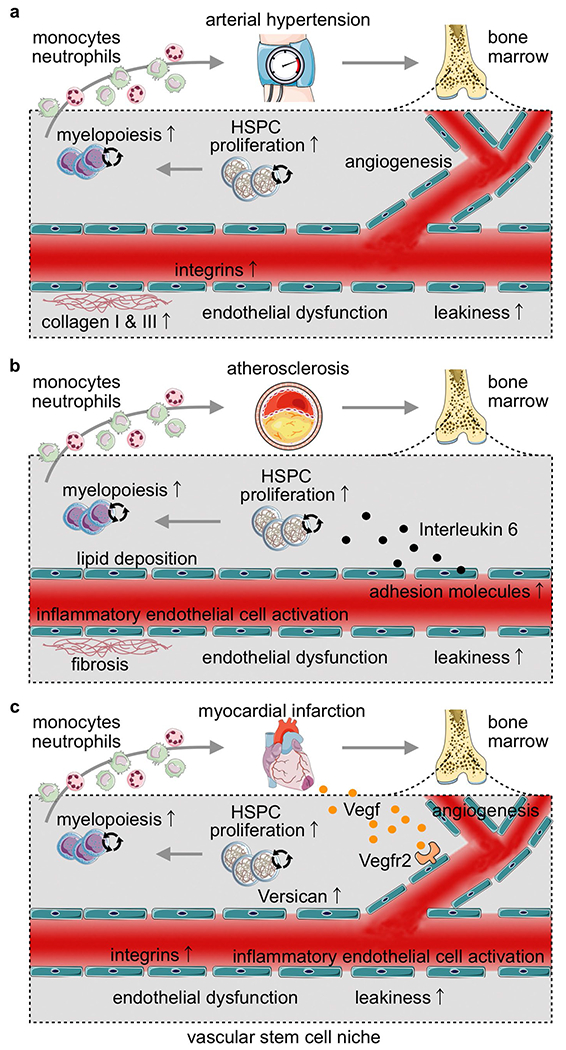
Summary cartoons, depicting the distinct bone marrow vascular phenotypes found in mice with arterial hypertension, atherosclerosis and myocardial infarction. **a**, In the bone marrow of mice with hypertension, we observed angiogenesis, increased perivascular collagen deposition and higher integrin expression. Functionally, bone marrow vessels were more leaky and the vasodilation response to acetylcholine was impaired, indicating endothelial dysfunction. These changes in the vascular niche were associated with increased hematopoietic progenitor proliferation and higher systemic numbers of innate immune cells. **b**, In the bone marrow of mice with atherosclerosis, we observed perivascular lipid deposition, vascular fibrosis, vascular leakiness, endothelial dysfunction, inflammatory endothelial cell activation, including increased Il6 expression. Il6 deletion from endothelial cells reduced hematopoiesis, indicating that the inflamed endothelium increases the production of immune cells in atherosclerosis. **c**, Mice with acute myocardial infarction exhibit Vegfa/Vefr2 dependent angiogenesis, which licensed emergency myelopoiesis (endothelial cell-specific Vegfr2 deletion dampened post-MI leukocytosis). In addition, bone marrow endothelial cells expressed more Versican, and deletion of the gene indicated that Versican also contributed to increased proliferation of hematopoietic stem and progenitor cells after MI. In the bone marrow of mice with acute MI we also observed endothelial dysfunction, vascular leakiness and inflammatory endothelial cell activation.

The hematopoietic marrow is a richly vascularized organ: the marrow’s vascular volume fraction is 21% in healthy mice, exceeding the heart’s fraction of 13% (ref. ^68^). Endothelial cells regulate HSPC proliferation and leukocyte transit^17^ and are thus an essential hematopoietic niche component. At the same time, endothelial cells are subject to and centrally involved in CVD and its complications. Our study addresses how the heart disease milestones hypertension, atherosclerosis and MI affect the bone marrow’s vascular health. Of note, these pathologies coexist in patients; for example, hypertension is a risk factor for atherosclerosis and MI rarely occurs without thrombotic complications of atherosclerotic plaque. Studying these conditions separately in mice allowed us to decipher their specific contributions to CVD-induced remodeling of the bone marrow’s vascular niche (Extended Data Fig. 8 and Supplementary Table 2). Endothelial dysfunction and microvascular leakage occurred in all three conditions, vascular fibrosis in hypertension and atherosclerosis, and angiogenesis in mice with hypertension and MI. The above adaptions of the vascular bone marrow niche can influence the bone marrow’s leukocyte output^15–17^. Integrin activation supports leukocyte adhesion and migration^45^, while a more permeable vascular wall promotes intravasation of newly produced cells. In addition, increased vascular leakage changes the hematopoietic niche’s microenvironment^14^ through expanded niche communication with circulating inflammatory or metabolic factors that may influence stem cell activity. The regional association of newly formed vessels with proliferating HSPCs suggests that post-MI angiogenesis expands vascular niche sites of leukocyte production, a speculation that is supported by our observation of blunted emergency hematopoiesis when angiogenesis is inhibited. Augmented levels of the canonical niche factor encoded by *Cxcl12* in *Cdh5*^CreERT2^;*Kdr*^fl/fl^ mice with MI may also contribute to lower myelopoiesis.

All three conditions transformed transcriptional programs of bone marrow endothelial cells, and the genetic manipulations that we induced to follow up on transcriptional changes highlight the importance of such phenotypic changes for increased inflammatory leukocyte supply. Whether IL-6 or versican, both of which have been shown to enhance cell proliferation^69,70^, act directly on HSPCs or alternatively indirectly via niche cells, remains to be formally investigated. Of note, HSPCs express the requisite receptors for IL-6 (IL-6 receptor) and versican (CD44, https://www.immgen.org/). Other open questions involve the specific molecular signals that induce endothelial cell phenotypes in mice with CVD, whether the observed pathways interact at the protein level and how non-endothelial hematopoietic niche cells respond to signals arising in the setting of cardiovascular pathologies. In particular, bone marrow fibroblasts, adipocytes and their mesenchymal progenitors are promising targets for future studies given that these cells are (1) systemically affected by CVD and metabolic disorders and (2) influential hematopoietic niche components.

While our work mostly focused on the anatomical and functional consequences of CVD for the vascular hematopoietic niche, it is worthwhile to consider its clinical implications, especially regarding therapeutic strategies aimed at reducing harmful leukocytosis and cardiovascular inflammation. Our observations of mice with atherosclerosis support that IL-6 receptor inhibition, which reduces inflammation in patients with atherosclerosis^71^, may act at least partially by reducing leukocyte production. Systemic inhibition of angiogenesis may disrupt infarct healing and formation of collaterals. If inhibition of bone marrow angiogenesis were to be harnessed therapeutically, organ-specific drug delivery could circumvent this issue, for instance, by using bone marrow-specific RNA interference^72^. The traditional cardiovascular continuum connects known risk factors such as hypertension and hypercholesterolemia to diseases of the myocardium and larger arteries. The current data enlarge this concept to include major vascular alterations that influence the bone marrow’s hematopoietic potential. These observations provide new mechanistic insight into how crosstalk between common risk factors, the heart and vasculature link leukocytes to cardiovascular conditions. At a conceptual level, these feedback loops indicate that studying the bone marrow vasculature in CVD will identify new pathways to therapeutically target escalating leukocytosis and systemic inflammation.

## Methods

### Human participants.

Control and patient bone marrow samples were obtained from the sternum or iliac crest following written informed consent in accordance with the Declaration of Helsinki and after approval by the institutional review boards of the University Hospital Germans Trias i Pujol (ICOR-2017-06, reference PI-17-176), the Amsterdam University Medical Center (NTR166, ISRCTN95796863) and Massachusetts General Hospital (2013P002438, 2018P001465). Patients in the atherosclerosis and hypertension cohort had coronary artery, cerebrovascular and/or peripheral artery disease combined with arterial hypertension. Sample collection in patients with MI was performed between day 3 and day 7 after ST-segment elevation MI. Tissue samples were immersed in PBS with 0.5% BSA and passed through a 40-μm cell strainer. Following centrifugation, the cell pellet was resuspended in 70% DMEM (Sigma-Aldrich), 20% FCS and 10% DMSO, transferred to cryovials and frozen. Additional cryopreserved bone marrow samples from healthy donors, obtained from iliac crest puncture, were purchased from Stemcell Technologies and HemaCare. Age distribution in controls and patient cohorts was as follows (mean ± s.d.): controls, 55.3 ± 13.5 years; hypertension, 69.8 ± 15.9 years; atherosclerosis and hypertension, 64.75 ± 7.4 years; MI, 61.1 ± 11.1 years.

### Flow cytometry of human bone marrow.

Cells were thawed at 37 °C, washed with sterile PBS and incubated with biotinylated anti-human lineage antibodies directed against CD2 (clone RPA-2.10, BioLegend), CD3 (HIT3a, 300304, BioLegend), CD4 (RPA-T4, BioLegend), CD7 (124-1D1, eBioscience), CD8a (RPA-T8, BioLegend), CD10 (SN5c, eBioscience), CD11b (ICRF44, BioLegend), CD14 (HCD14, BioLegend), CD19 (HIB19, BioLegend), CD20 (2H7, eBioscience), CD56 (HCD56, BioLegend) and GPA (HIR2, BioLegend) and LIVE/DEAD Fixable Aqua Dead Cell Stain (L-34957, Life Technologies). For gating on hematopoietic progenitor cells, this was followed by secondary staining with anti-human CD34–APC (8G12, BD Biosciences), anti-CD38–PE/Cy7 (HIT2, BioLegend), anti-CD90–FITC (5E10, BD Biosciences), anti-CD45RA-PB (MEM-56, Thermo Fisher Scientific), anti-CD123–PE (7G3, BD Biosciences) and streptavidin-APC/Cy7 (BioLegend). Intracellular staining with anti-human Ki-67–BV605 antibody (BioLegend) was performed using the BD Cytofix/Cytoperm Fixation/Permeabilization kit. Samples were run on an LSR II flow cytometer (BD Biosciences), and recorded events were analyzed with FlowJo 10 software (BD). Individual fluorescence-minus-one controls were used to determine gating. HSCs were identified as lineage^−^CD34^+^CD38^−^CD45RA^lo^CD90^+^ cells, CMPs as lineage^−^CD34^+^CD38^int^CD45 RA^−^CD123^int^ cells and GMPs as lineage^−^CD34^+^CD38^int^CD45RA^+^CD123^int^ cells.

### Mouse models.

Wild-type C57BL/6J, *Apoe*^tm1Unc^ (*Apoe*^−/−^), BPN/3J, BPH/2J, B6.Cg-*Gt*(*ROSA*)*26Sor*^tm6(*CAG*-ZsGrsen1)Hze^/J (*Rosa26*^ZsGreen^), B6.Cg-*Commd10*^Tg(*Vav1*-iCre)A2Kio^/J (*Vav1*^Cre^), *Kdr*^tm2Sato^/J (*Kdr*^*fl/fl*^) and B6.SJL-*Ptprc*^a^ *Pepc*^b^/BoyJ (CD45.1) mice were purchased from Jackson Laboratory. *Cdh5*(PAC)-CreERT2 (*Cdh5*^CreERT2^) mice were purchased from Taconic. *Vcan*^tm1.1Hwat^ (*Vcan*^fl/fl^) mice were provided by H. Watanabe (Institute for Molecular Science of Medicine, Aichi Medical University, Aichi, Japan)^73^. *Il6*^tm1.1Jho^ (*Il6*^fl/fl^) mice were provided by J. Hidalgo (Institute of Neurosciences and Department of Cellular Biology, Physiology and Immunology, Universitat Autònoma de Barcelona, Barcelona, Spain). *Apln*^tm1.1(Cre/ERT2)Bzsh^ (*Apln*^CreER^) mice were provided by B. Zhou (State Key Laboratory of Cell Biology, CAS Center for Excellence in Molecular Cell Science, Institute of Biochemistry and Cell Biology, Chinese Academic of Sciences, Shanghai, China)^42^. Atherosclerotic *Apoe*^−/−^ mice were fed a western-type diet (TD.88137, Envigo) for 10 weeks. Atherosclerosis in *Cdh5*^CreERT2^;*Il6*^fl/fl^ and *Cdh5*^CreERT2^ controls was induced by a single i.v. injection of 10^12^ genomic copies of AAV 2/8 encoding hPCSK9 (ref. ^74^), as calculated by ITR primer titers. Mice were placed on an atherogenic high-cholesterol diet for 12 weeks (Research Diets). The *PCSK9* plasmid pAAV/D377Y-mPCSK9 was provided by Addgene^75^. Hemizygous *Cdh5*^CreERT2^ mice received intraperitoneal injections of 75 mg per kg tamoxifen dissolved in corn oil (both from Sigma-Aldrich). A total of six injections were performed. Cre littermate controls were subjected to the same injections. InVivoMAb anti-mouse VEGFR2 antibody (clone DC101, Bio X Cell) was injected intraperitoneally 2 h before and 24 and 48 h after MI at a dose of 800 μg. When appropriate, animals were randomly assigned to experimental groups. All protocols were approved by the Institutional Animal Care and Use Committee at Massachusetts General Hospital, and all animal experiments were performed in compliance with relevant ethical regulations.

### Mouse surgical interventions.

For Ang-II treatment, ALZET osmotic minipumps (Durect) were implanted subcutaneously in mice under anesthesia with isoflurane. To maintain an application rate of Ang-II at 490 ng min^−1^ per kg (A9525, Sigma-Aldrich) for 8 weeks, minipump model 1004 was implanted first and then exchanged for model 2006 after 3 weeks^76,77^. For PBS or VEGFA (450-32, PeproTech) treatment, ALZET osmotic minipump model 1003D (Durect) was implanted into the peritoneal cavity. Transverse aortic constriction was induced as previously described^78^. Sham-operated animals were subjected to the same procedure, including aortic arch mobilization, but without ligation. Mice were analyzed 8 weeks after surgery. Permanent coronary artery ligation to induce MI was performed as described before^37^. For all surgical interventions, animals were given buprenorphine (0.1 mg per kg subcutaneously) before and every 12 h for the first 72 h after surgery.

### Bone marrow transplantation.

To obtain bone marrow chimeric mice, C57BL/6J mice were lethally irradiated (970 cGy). BMNCs were obtained from femora of *Vav1*^Cre^ or *Vav1*^Cre^;*Kdr*^fl/fl^ mice. One day after irradiation, 3–5 × 10^6^ BMNCs were transplanted into the irradiated recipients via retro-orbital injection.

### Histology and immunostaining.

Femurs were dissected, fixed in 4% paraformaldehyde and decalcified in 0.5 M EDTA at 4 °C for 2 weeks. Femurs were then transferred to 30% sucrose–PBS at 4 °C for 2 d, incubated in equal-volume OCT-30% sucrose overnight and embedded in OCT (Sakura Finetek). For collagen staining, sections were incubated with primary antibodies against collagen I (AB765P, Sigma-Aldrich), collagen type III (ab7778, Abcam) or collagen type IV (AB756P, Sigma-Aldrich), followed by treatment with peroxidase-conjugated anti-rabbit antibody (ab205718, Abcam) and 3,3’-diaminobenzidine (D8001, Sigma-Aldrich). Femur sections were stained with antibodies against EMCN (AF4666, Novus Biologicals) and laminin (ab11575, Abcam, both at 1:200). Primary antibodies were labeled with AF546-conjugated and AF680-conjugated secondary antibodies (Thermo Fisher Scientific). Musculus quadriceps femoris samples were stained with anti-mouse CD31 antibody (MEC13.3, BD Biosciences). Fluorescent images were acquired with a Nikon 80i microscope (Nikon). Light microscopy images were captured using a digital slide scanner, NanoZoomer 2.0RS (Hamamatsu), and analyzed using ImageJ software.

### Flow cytometry and cell sorting of murine samples.

To assess bone marrow hematopoietic cells, mice were anesthetized and flushed with 20 ml PBS. A single-cell suspension was created by passing the bone marrow through a 40-μm cell strainer. Spleen tissue was minced and plunged through a 40-μm cell strainer before red blood cell lysis was performed with RBC lysis buffer (BioLegend). Peripheral blood was collected by retro-orbital bleeding using heparinized capillary tubes (BD). For bone marrow stromal cells, bone marrow was flushed out and enzymatically digested with 1 mg ml^−1^ collagenase type IV (Worthington Biochemical Corporation) and 2 mg ml^−1^ dispase II (Sigma-Aldrich).

Bone marrow and spleen endothelial cells were stained with anti-mouse TER-119–BV605 (clone TER-119, BioLegend), anti-CD41–PerCP/Cy5.5 (MWReg30, BioLegend), anti-CD45.2–FITC (104, BD Biosciences), anti-CD31–PE (MEC13.3, BioLegend) and anti-SCA-1–PE/Cy7 (D7, BioLegend) antibodies and DAPI. To differentiate sinusoidal and arteriolar endothelial cells, 30 μl anti-mouse podoplanin–APC antibody (8.1.1, BioLegend) was injected i.v. 30 min before cell collection. To assess NO levels, cell suspensions were pre-incubated with the NO sensor 4-amino-5-methylamino-2′,7′-difluorofluorescein diacetate (DAF-FM diacetate, Thermo Fisher Scientific). Following antibody staining for bone marrow endothelial cells (anti-CD45–BV605 antibody, clone 30-F11, BioLegend), DAF-FM fluorescence was detected in the FITC channel. For identification of non-endothelial stromal cells, the following staining panel was used: anti-mouse TER-119-APC/Cy7 (TER-119, BioLegend), anti-CD41–PerCP/Cy5.5 (MWReg30, BioLegend), anti-CD45–BV605 (30-F11, BioLegend), anti-CD31–PE (MEC13.3, BioLegend), anti-SCA-1–PE/Cy7 (D7, BioLegend) and anti-CD106–AF647 (429, BioLegend) antibodies. For identification of HSPCs, isolated bone marrow cells were first stained with biotin-conjugated anti-mouse lineage antibodies directed against CD3 (clone 145-2C11, BioLegend), CD4 (GK1.5, BioLegend), CD8a (53-6.7, BioLegend), CD49b (DX5, BioLegend), CD90.2 (30-H12, BioLegend), CD19 (6D5, BioLegend), B220 (RA3-6B2, BioLegend), NK1.1 (PK136, BioLegend), TER-119 (TER-119, BioLegend), CD11b (M1/70, BioLegend), CD11c (N418, BioLegend) and GR1 (RB6-8C5, BioLegend). This was followed by a second staining with (1) anti-CD16/32–BV711 (clone 93, BioLegend), anti-CD34–FITC (RAM34, BD Biosciences), anti-CD48–AF700 (HM48-1, BioLegend), anti-CD115–BV421 (AFS98, BioLegend), anti-CD150–PerCP/Cy5.5 (TC15-12F12.2, BioLegend), anti-c-Kit–PE/Cy7 (2B8, BioLegend), anti-SCA-1–BV605 (D7, BioLegend), anti-Flk2–PE (A2F10, eBioscience) and APC/Cy7–streptavidin (405208, BioLegend) antibodies or (2) anti-c-Kit-PE/Cy7 (2B8, BioLegend), anti-SCA-1–BV421 (D7, BioLegend), anti-Flk2–PE (A2F10, eBioscience), anti-CD48–AF700 (HM48-1, BioLegend), anti-CD150-PerCP/Cy5.5 (TC15-12F12.2, BioLegend), anti-CD34–FITC (RAM34, BD Biosciences), anti-CD16/32–APC/Cy7 (93, BioLegend), anti-CD115–PerCP/eFlour710 (AFS98, eBioscience) and streptavidin–BV605 (BioLegend) antibodies. For blood and bone marrow leukocyte staining, cells were stained with anti-B220–PE/Cy7 (clone RA3-6B2, BioLegend), anti-CD19–PE/Cy7 (6D5, BioLegend), anti-CD3–PE (17A2, BioLegend), anti-CD45–AF700 (30-F11, BioLegend), anti-Ly6C–BV605 (HK1.4, BioLegend), anti-Ly6G–FITC (1A8, BioLegend), anti-CD11b–APC (M1/70, BioLegend), anti-NK1.1–APC/Cy7 (PK136, BioLegend) and anti-CD115–BV421 (AFS98, BioLegend) antibodies. Cells were further stained with the APC BrdU Flow kit (BD Biosciences) or anti-Ki-67–eFluor660 antibody (SolA15, eBioscience). BrdU was administered via intraperitoneal injection 24 h before killing mice. To assess blood leukocyte CD45.1–CD45.2 chimerism, the following staining panel was used: anti-mouse Ly6G–FITC (1A8, BioLegend), anti-CD11b–PE (M1/70, BioLegend), anti-CD3–PerCP/Cy5.5 (145-2C11, BD Biosciences), anti-CD115–BV421 (AFS98, BioLegend), anti-Ly6C–BV605 (HK1.4, BioLegend), anti-CD8a-BV711 (53-6.7, BioLegend), anti-CD4–AF700 (GK1.5, BioLegend), anti-CD45.2–APC (104, BioLegend), anti-CD45.1–PE/Cy7 (A20, BD Biosciences), anti-CD19–APC/Cy7 (6D5, BioLegend), anti-B220-APC/Cy7 (RA3-6B2, BioLegend) and anti-NK1.1–APC/Cy7 (PK136, BioLegend) antibodies.

Bone marrow and spleen endothelial cells were identified as TER-119^−^CD41^−^CD45^−^CD31^hi^SCA-1^+^ cells. Arteriolar endothelial cells were identified as TER-119^−^CD41^−^CD45^−^CD31^hi^SCA-1^+^PDPN^−^ cells, and sinusoidal endothelial cells were identified as TER-119^−^CD41^−^CD45^−^CD31^hi^SCA-1^+^PDPN^+^ cells. Bone marrow MSCs were identified as TER-119^−^CD41^−^CD45^−^CD31^−^SCA-1^−^CD106^+^ cells and fibroblasts as TER-119^−^CD41^−^CD45^−^CD31^−^CD106^−^SCA-1^+^ cells. Skeletal muscle endothelial cells were identified as CD45^−^CD31^+^ cells^79^. LSK were identified as Lin^−^c-Kit^+^SCA-1^+^ cells and SLAM–LSK as Lin^−^c-Kit^+^SCA-1^+^CD150^+^CD48^−^ cells^80,81^. CMPs were identified as Lin^−^c-Kit^+^SCA-1^−^CD16/32^int^CD34^+^ cells and GMPs as Lin^−^c-Kit^+^SCA-1^−^CD16/32^hi^CD34^+^ cells^82^. Monocytes were identified as CD45^+^NK1.1^−^CD3^−^CD19^−^B220^−^CD11b^+^SSC-A^lo^CD115^+^Ly6G^−^ cells and neutrophils as CD45^+^NK1.1^−^CD3^−^CD19^−^B220^−^CD11b^+^SSC-A^lo^CD115^−^Ly6G^+^ cells. Cell suspensions were sorted with a FACSAria II (BD Biosciences).

### RNA isolation and real-time PCR.

RNA was isolated from tissue samples with the RNeasy Mini kit (Qiagen) following the manufacturer’s instructions. RNA was extracted using the NucleoSpin RNA XS Kit for RNA-seq (Takara Bio) or the ARCTURUS PicoPure RNA Isolation kit (Thermo Fisher Scientific). The High-Capacity RNA-to-cDNA kit (Applied Biosystems) was used for reverse transcription. TaqMan gene expression assays were used to quantify target genes using TaqMan Fast Universal PCR Master Mix (Applied Biosystems) and primers for *Cxcl12* (Mm00445553_m1), *Vcam1* (Mm01320970_m1), *Kitl* (Mm00442972_m1), *Angpt1* (Mm00456503_m1), *Il6* (Mm00446190_m1), *Vegfa* (Mm00437306_m1) and *Vcan* (Mm01283063_m1, all FAM–MGB, Thermo Fisher Scientific) as well as *Gapdh* (Mm99999915_g1, VIC–MGB, Thermo Fisher Scientific). Samples were run on a 7500 Real-Time PCR system (Applied Biosystems), and target gene expression was normalized to *Gapdh* expression.

### Enzyme-linked immunosorbent assay.

Blood was centrifuged for 20 min at 3,000*g*, and plasma was gathered from the supernatant. Bone marrow interstitial fluid was obtained from femora of mice perfused with 20 ml PBS. Femora were cut with scissors and centrifuged at 16,000*g* for 2 min. The bone marrow was then vortexed for 5 s and centrifuged at 5,000*g* for 5 min. Quantitative measurement of VEGF was performed according to the manufacturer’s protocol using the Mouse VEGF Quantikine ELISA kit (R&D Systems). The Mouse IL-6 Quantikine ELISA kit (R&D Systems) was used for assessment of IL-6 levels.

### Colony-forming unit assay.

Colony-forming unit assays were performed using a semi-solid methylcellulose matrix supplemented with cytokines and growth factors (MethoCult GF M3434, Stemcell Technologies) following the manufacturer’s manual. Whole blood (50 μl) was processed, and the cell-containing medium was subsequently plated into two 35-mm cell culture dishes and incubated for 10–14 d.

### Competitive bone marrow repopulation assay.

Donor mice were perfused with 20 ml PBS before one femur was excised. Bone marrow was flushed out, and 5 × 10^5^ BMNCs were obtained after plunging the tissue through a 40-μm cell strainer. BMNCs from mice in the respective experimental conditions (CD45.2) were mixed with BMNCs from naive CD45.1 animals before being injected into lethally irradiated CD45.2 recipients (970 cGy).

### Confocal microscopy.

Confocal microscopy of the skull and femur bone marrow was performed with an IV100 microscope (Olympus)^44^. The field of view at 4× magnification covers 2,290 μm by 2,290 μm, and that at 20× covers 458 μm by 458 μm with a resolution of 512 by 512 pixels. *z*-stack images were acquired at 2-μm steps. In anesthetized mice, the calvarial bone was exposed, and mice were positioned in a stereotactic holder. OsteoSense 750 was injected i.v. 24 h before imaging (4 nmol per mouse, PerkinElmer). IntegriSense 680 was administered i.v. 24 h before imaging (2 nmol per mouse, PerkinElmer). To delineate blood vessels, 30 μl anti-mouse SCA-1–PE antibody (clone D7, BioLegend) and 30 μl anti-mouse CD31–PE antibody (MEC13.3, BioLegend) were injected i.v. To investigate vascular leakage, we followed a single plane through the center of the bone marrow cavity over 30 s with time-lapse acquisition of 1.2 s per image before and after an i.v. bolus of 2.5 mg albumin–FITC (2.5 mg per mouse, Sigma-Aldrich). During in vivo imaging of blood flow dynamics in bone marrow microvessels, the injected albumin-FITC labels blood plasma yet is excluded from blood cells, which appear as dark objects in bright fluorescent plasma^83^. Line scans of cell movement were performed in *n* = 2–6 arterioles per animal before and during i.v. infusion with 10^−6^ M acetylcholine. LSK isolated by flow cytometry were labeled ex vivo with Vybrant DiD Cell-Labeling Solution (Thermo Fisher Scientific) according to the manufacturer’s protocol. Mice were injected i.v. with 75,000 DiD-labeled LSK. For ex vivo confocal imaging of femoral bone marrow, mice were injected with OsteoSense 750 (4 nmol per mouse, PerkinElmer) 24 h before imaging. Before collecting the femur (30 min), mice were injected i.v. with 30 μl anti-mouse CD31–PE (MEC13.3, BioLegend), 30 ol anti-mouse SCA-1–AF647 (D7, BioLegend) and 50 μl anti-mouse EMCN–FITC (V.7C7, Santa Cruz Biotechnology) antibodies. Femurs were fixed in 4% paraformaldehyde, embedded in OCT and stained with anti-mouse CD11b–APC antibody (M1/70, BioLegend) for 30 min at room temperature and imaged by confocal microscopy.

### Positron emission tomography-magnetic resonance imaging.

Dynamic contrast-enhanced MRI relied on BSA labeled with rhodamine B and gadopentetic acid (RhoB–albumin–GdDTPA; 82 kDa, SyMO-Chem). For PET imaging, NODAGA–RGD trifluoroacetate (cyclo(–Arg–Gly–Asp-d–Tyr–Lys(NODAGA)–)) was acquired from ABX Advanced Biochemical Compounds. The ^68^GaCl_3_ compound was obtained from a ^68^Ge/^68^Ga generator (ITG) and eluted with 0.1 M HCl. NODAGA–RGD (30 μg, 30.7 μmol) was dissolved with 0.1 M ammonium acetate buffer (100 μl, pH 6) and combined with ^68^GaCl_3_. The labeling mixture was heated at 80 °C for 10 min on a thermomixer at 900 r.p.m. The mixture was loaded on a Sep-Pak C18 Plus cartridge (WAT020515, Waters), and the labeled product was eluted with 1.5 ml ethanol followed by drying at 65 °C with a gentle argon stream. Analytical radio–HPLC demonstrated >99% radiochemical purity using a 1260 Infinity system (Agilent Technologies). Specific activity was ~8.02 GBq μmol^−1^ with a radiochemical yield of ~72%.

Magnetic resonance and PET–CT imaging were performed sequentially^44^. Mice were imaged in a 4.7-tesla PharmaScan (Bruker) with a mouse volume coil (Rapid Biomedical). *T*_2_-weighted MRI was followed by albumin-based dynamic contrast-enhanced (DCE)-MRI. A RARE sequence was first acquired with the following parameters for *T*_2_-weighted imaging with fat suppression: TE, 40 ms; TR, 4,000 ms; echo spacing, 10 ms; RARE factor, 10; averages, 8; FOV, 40 × 40 mm; matrix, 256 × 256; slice thickness, 1 mm; 20 axial slices; spatial resolution, 0.156 × 0.156 × 1 mm^3^ per voxel; acquisition time, 13 min 20 s. For macromolecular DCE-MRI, a bolus of RhoB–albumin–GdDTPA (10 mg per mouse, relaxivity of 145 mM s^−1^, SyMO-Chem) was injected i.v. Following a series of variable flip-angle precontrast *T*_1_-weighted fast low-angle shots to determine the endogenous precontrast *R*_1_ values, dynamics of labeled albumin were followed every 25 s for 4 min after contrast administration with 2D fast low-angle shot images covering the entire femur. The following imaging parameters were used: precontrast flip angles, 2°, 6°, 9°, 15°, 25°, 50°, 70°; post-contrast flip angle, 50°; TR = 25 ms; TE = 2.748 ms; number of averages, 2; FOV, 20 × 20 mm; 1 slice; slice thickness, 1.2 mm; spatial resolution, 0.078 × 0.078 × 1.2 mm^3^ per voxel; temporal resolution, 10 s 200 ms. After, mice were transferred into an Inveon PET–CT (Siemens). The ^68^Ga-NODAGA–RGD tracer was injected i.v. 120 min before PET imaging^44^. PET acquisition time was 30 min. A 3D ordered subset expectation maximization (OSEM) with maximum a posteriori (MAP) algorithm was used with two OSEM iterations and 18 MAP iterations for PET reconstruction.

### Scintillation counting and autoradiography.

Direct gamma counting of various bones was performed using a gamma counter (1480 Wizard 3” Gamma Counter, PerkinElmer). For autoradiography, femurs from both controls and mice with MI were exposed on a phosphoimager (Amersham Typhoon 9410, GE Healthcare).

### RNA sequencing.

To identify pathways associated with bone marrow alterations in CVD, we performed transcriptional profiling using whole-transcriptome sequencing (RNA-seq) in flow cytometry-isolated endothelial cells (bone marrow endothelial cells) and hematopoietic progenitor cells (LSK). Samples were lineage depleted by staining with PE-conjugated anti-mouse antibodies directed against CD4, CD8, CD19, B220, NK1.1, TER-119, CD11b, CD11c, Ly6G and CD127, followed by incubation with anti-PE microbeads (130-097-054, Miltenyi Biotec) and subsequent magnetic separation using LD columns (130-042-90, Miltenyi Biotec). RNA was isolated from sorted cells using the NucleoSpin RNA XS kit (740902.50, Takara Bio). For bone marrow endothelial cells, total isolated RNA was subjected to polyA selection, followed by NGS library construction using the NEBNext Ultra Directional RNA Library Prep kit for Illumina (New England Biolabs). For LSK, NGS library construction was performed using the Clontech SMARTer Ultra Low Input RNA kit for Illumina (634936, Takara Bio USA). Sequencing was performed on an Illumina HiSeq 2500 instrument (Illumina), resulting in 29–39 million single-end 50-bp reads per sample. Library preparation and sequencing were performed by the MGH NextGen Sequencing Core.

### Evans Blue assay.

To assess blood vessel permeability by short-term protein extravasation in the bone marrow, a modified Evans Blue assay was used. Mice received i.v. 100 μl Evans Blue (10 mg ml^−1^ in 0.9% sodium chloride). After 5 min, animals were perfused with 20 ml PBS. One femur was collected and cut, and the bone marrow was isolated by centrifugation at 16,000*g* for 2 min. The bone marrow was then homogenized in 100 μl PBS and centrifuged at 5,000*g* for 5 min. Bone marrow interstitial fluid was collected from the supernatant, and absorbance was assessed at 620 nm on a Spark microplate reader (Tecan). Absolute Evans Blue concentrations were calculated from a standard curve.

### Confocal imaging analysis.

Images were processed and analyzed using AngioTool 0.5, ImageJ version1.51r and MATLAB R2015b (MathWorks). To quantify the number of vessel branch points in the calvarial bone marrow, maximum intensity projections from *z* stacks containing 21 images (total depth of 40 μm) were analyzed with AngioTool 0.5, applying the same parameters for all datasets without correcting for potential blood vessel superimposition^84^. For IntegriSense (on *z* stacks) and vascular leak (on single slices) analyses, a bone marrow ROI was selected according to the OsteoSense signal, and an ROI for the background was picked within an osseous region^44^. For IntegriSense, maximum intensity projections of *z* stacks with 16 slices were used to obtain target-to-background ratios. Calculating vascular leakage relied on the signal intensity derived from the first image after albumin–FITC injection, which was used to generate a thresholded image of the bone marrow vasculature. The thresholded area was used to segment the signal intensity derived from albumin ‘in’ and ‘out’ of the vasculature. The ratio between these two intensities (*I*_out_/*I*_in_) per time unit denotes vascular leakiness at the given location^44^. To quantify blood flow velocity and endothelial function, line scan analyses were performed to trace red blood cell displacement and to calculate the velocity of erythrocytes based on the distance they were displaced per time unit^14,83^. Using the difference in blood flow velocities measured before and during acetylcholine injection, Δvelocity was calculated^85^. Analyzing vessel proximity to CD11b^+^ myeloid cells relied on three to four images of an identified vessel of interest in *a z* stack. Images were thresholded for CD11b–APC^+^ pixels, and distances to the nearest CD31^+^ SCA-1^+^ and ZsGreen^+^ blood vessel were calculated excluding osseous areas^86^. ROI for sprout analysis were analyzed in femoral bone marrow views of 100 μm × 100 μm. To quantify the number of sprouts from a maximal intensity projection with 13–17 slices, sprouts protruding >8 μm from a blood vessel were manually counted.

### MRI and PET data analysis.

For albumin-based DCE-MRI, femoral MRI data were analyzed on a pixel-by-pixel basis with MATLAB R2015b software (MathWorks). Calculation of RhoB–albumin–GdDTPA signal for the selected slice in dynamic datasets was based on the relaxivity of RhoB–albumin–GdDTPA as described previously^44,68^. ROI were drawn for the entire femur. RhoB–albumin–GdDTPA concentrations in the ROI were normalized to the concentration in blood (calculated from ROI in the vena cava). Precontrast and post-contrast mean femoral *R*_1_ values were calculated using variable flip-angle (*α*) data by nonlinear best fit. The fractional blood volume was derived by extrapolating the linear regression of the normalized concentrations to the time of contrast administration. Fractional blood volume was 20.8% ± 4.4% for healthy bone marrow (*n* = 6 mice). From the slope of normalized concentration values, the rate of contrast accumulation or permeability × surface area product (permeability, min^−1^) was derived from a linear regression of the normalized concentrations over time. The permeability quantified with RhoB–albumin–GdDTPA shows albumin extravasation from blood vessels and its accumulation in the tissue. Femoral parametric permeability maps were projected to indicate mean values. PET standardized uptake values were obtained as described previously^87^. Image co-registration of PET and MRI datasets was carried out as described previously^87^.

### RNA-seq analysis.

The STAR aligner 2.7.3a^88^ was used to align sequencing reads to the mouse reference genome GRCm38.p6. Read counts per gene were obtained from STAR’s ‘quantMode’ with the ‘GeneCounts’ option using mouse genome annotations from Ensembl database release 98 (ref. ^89^). Differential gene expression was tested with likelihood-ratio tests based on the generalized linear model implementation of edgeR 3.28.0 (ref. ^90^). GSEA was performed with the Preranked tool from GSEA 4.0.3 (refs. ^91,92^) using gene set collections in the Molecular Signatures Database release 7.0. The log_2_ (fold change) values obtained from differential expression analysis were used as the ranking metric, with the ‘weighted’ option for the enrichment score. Enriched gene ontology biological processes were obtained with clusterProfiler^93^.

### Statistical tests.

Statistical analyses were performed using GraphPad Prism 8 (GraphPad Software). Results are reported as mean ± s.e.m. Normality was assessed using D’Agostino–Pearson omnibus normality test. For a two-group comparison, normally distributed datasets underwent a parametric two-tailed Welch’s *t*-test, whereas non-normally distributed data were evaluated with a nonparametric two-tailed Mann-Whitney test.

### Reporting Summary.

Further information on research design is available in the Nature Research Reporting Summary linked to this article.

## Extended Data

## Supplementary Material

In vivo microscopic time-lapse imaging of vascular leakage in calvarial bone marrow of control mice.

In vivo microscopic time-lapse imaging of vascular leakage on day 2 after myocardial infarction

In vivo microscopic time-lapse imaging of vascular leakage in mice 2 days post myocardial infarction

Supplementary Tables

1752094_RS

## Data Availability

Data were deposited in NCBI’s Gene Expression Omnibus and are accessible through GEO series accession number GSE144498 (bone marrow endothelial cell RNA-seq).
