# Supplementary material for: Bone marrow endothelial dysfunction promotes myeloid cell expansion in cardiovascular disease
Source: Nat Cardiovasc Res. Author manuscript; Available in PMC 2022 Jun 22. (PMC9216333; doi:10.1038/s44161-021-00002-8)
Supplement: 1752094_RS [file NIHMS1752094-supplement-1752094_RS.pdf]

nature portfolio

Corresponding author(s): Matthias Nahrendorf

Last updated by author(s): Oct 24, 2021

Reporting Summary

Nature Portfolio wishes to improve the reproducibility of the work that we publish. This form provides structure for consistency and transparency in reporting. For further information on Nature Portfolio policies, see our Editorial Policies and the Editorial Policy Checklist.

Statistics

For all statistical analyses, confirm that the following items are present in the figure legend, table legend, main text, or Methods section.

n/a	Confirmed	
☐	☒	The exact sample size (*n*) for each experimental group/condition, given as a discrete number and unit of measurement
☐	☒	A statement on whether measurements were taken from distinct samples or whether the same sample was measured repeatedly
☐	☒	The statistical test(s) used AND whether they are one- or two-sided<br>*Only common tests should be described solely by name; describe more complex techniques in the Methods section.*
☐	☒	A description of all covariates tested
☐	☒	A description of any assumptions or corrections, such as tests of normality and adjustment for multiple comparisons
☐	☒	A full description of the statistical parameters including central tendency (e.g. means) or other basic estimates (e.g. regression coefficient) AND variation (e.g. standard deviation) or associated estimates of uncertainty (e.g. confidence intervals)
☒	☐	For null hypothesis testing, the test statistic (e.g. *F*, *t*, *r*) with confidence intervals, effect sizes, degrees of freedom and *P* value noted<br>*Give P values as exact values whenever suitable.*
☒	☐	For Bayesian analysis, information on the choice of priors and Markov chain Monte Carlo settings
☒	☐	For hierarchical and complex designs, identification of the appropriate level for tests and full reporting of outcomes
☒	☐	Estimates of effect sizes (e.g. Cohen's *d*, Pearson's *r*), indicating how they were calculated

Our web collection on statistics for biologists contains articles on many of the points above.

Software and code

Policy information about availability of computer code

Data collection	Flow cytometry data were acquired on an LSRII flow cytometer equipped with FACS Diva 6.1 software (BD Biosciences, San Jose, CA, USA). Light microscopy images were captured on a NanoZoomer 2.0RS (Hamamatsu, Shizuoka, Japan). Immunofluorescence images were taken with a Nikon 80i (Nikon, Tokyo, Japan). qPCR data were acquired on a 7500 Real-Time PCR system (Applied Biosystems, Foster City, CA, USA). PET/CT imaging was performed with an Inveon PET/CT system using Inveon Research Workplace 3.0 and Inveon Acquisition Workplace 1.5.028 software (Siemens, Malvern, PA, USA). Confocal microscopy was executed with an IV100 microscope and IV10-ASW 01.01.00.05 software (Olympus, Tokyo, Japan). MRI was performed with a 4.7 Tesla PharmaScan and Paravision 6.0.1 software (Bruker, Billerica, MA, USA). Scintillation counting was done with a 1480 Wizard 3” Gamma Counter (PerkinElmer, Waltham, MA, USA), and autoradiography with an Amersham Typhoon 9410 phosphoimager (GE Healthcare, Chicago, IL, USA). RNA-seq DNA libraries were sequenced on a HiSeq 2500 instrument (Illumina, San Diego, CA, USA).
Data analysis	Flow cytometry data were analyzed with FlowJo 10 software (BD, Franklin Lakes, NJ, USA). Light microscopy and immunofluorescence images were analyzed using ImageJ 1.51r software. 3D reconstruction of PET, CT, and MRI datasets was done with Amira 5.3.2 software (Thermo Fisher Scientific, Waltham, MA, USA). Confocal microscopy images were analyzed using AngioTool 0.5, ImageJ 1.51r, and MATLAB R2015b software (Mathworks, Natick, MA, USA). PET/MRI data were analyzed with MATLAB R2015b software. RNA-seq data were processed and analyzed using STAR aligner 2.7.3a, edgeR 3.28.0, GSEA 4.0.3., and clusterProfiler. GraphPad Prism 8 software (GraphPad, San Diego, CA, USA) was used for statistical analysis.

For manuscripts utilizing custom algorithms or software that are central to the research but not yet described in published literature, software must be made available to editors and reviewers. We strongly encourage code deposition in a community repository (e.g. GitHub). See the Nature Portfolio guidelines for submitting code & software for further information.

Data

Policy information about availability of data

All manuscripts must include a data availability statement. This statement should provide the following information, where applicable:

- Accession codes, unique identifiers, or web links for publicly available datasets
- A description of any restrictions on data availability
- For clinical datasets or third party data, please ensure that the statement adheres to our policy

RNA-seq data were deposited in NCBI's Gene Expression Omnibus and are accessible through GEO Series accession number GSE144498 (bone marrow endothelial cells).

Field-specific reporting

Please select the one below that is the best fit for your research. If you are not sure, read the appropriate sections before making your selection.

☒ Life sciences ☐ Behavioural & social sciences ☐ Ecological, evolutionary & environmental sciences

For a reference copy of the document with all sections, see nature.com/documents/nr-reporting-summary-flat.pdf

Life sciences study design

All studies must disclose on these points even when the disclosure is negative.

Sample size	Sample size was approximated based on prior experiments and available data without statistical predetermination.
Data exclusions	No data points were excluded.
Replication	Experiments were reproduced at least once to confirm the obtained results.
Randomization	Animals were randomly assigned to experimental groups.
Blinding	Investigators were blinded where appropriate.

Reporting for specific materials, systems and methods

We require information from authors about some types of materials, experimental systems and methods used in many studies. Here, indicate whether each material, system or method listed is relevant to your study. If you are not sure if a list item applies to your research, read the appropriate section before selecting a response.

Materials & experimental systems

n/a	Involved in the study	
☐	☒	Antibodies
☒	☐	Eukaryotic cell lines
☒	☐	Palaeontology and archaeology
☐	☒	Animals and other organisms
☐	☒	Human research participants
☒	☐	Clinical data
☒	☐	Dual use research of concern

Methods

n/a	Involved in the study	
☒	☐	ChIP-seq
☐	☒	Flow cytometry
☒	☐	MRI-based neuroimaging

Antibodies

Antibodies used

Antibodies for flow cytometry:
Anti-human CD2 (clone RPA-2.10, catalog number 300204, BioLegend), CD3 (HIT3a, 300304, BioLegend), CD4 (RPA-T4, 300504, BioLegend), CD7 (124-1D1, 13-0079-82, eBioscience), CD8a (RPA-T8, 301004, BioLegend), CD10 (SN5c, 13-0108-82, eBioscience), CD11b (ICRF44, 301304, BioLegend), CD14 (HCD14, 325624, BioLegend), CD19 (HIB19, 302204, BioLegend), CD20 (2H7, 13-0209-82, eBioscience), CD56 (HCD56, 318320, BioLegend), GPA (HIR2, 306618, BioLegend), CD34-APC (8G12, 340667, BD Biosciences), CD38-PE/Cy7 (HIT2, 303516, BioLegend), CD90-FITC (5E10, 555595, BD Biosciences), CD45RA-PB (MEM-56, MHCD45RA28, Thermo Fisher Scientific), CD123-PE (7G3, 554529, BD Biosciences), Ki-67-BV605 (Ki-67, 350522, BioLegend).
Anti-mouse Ter119-BV605 (clone TER-119, catalog number 116239, BioLegend), CD41-PerCP/Cy5.5 (MWReg30, 133918, BioLegend), CD45.2-FITC (104, 553772, BD Biosciences), CD31-PE (MEC13.3, 102508, BioLegend), Sca-1-PE/Cy7 (D7, 108114, BioLegend), Podoplanin-APC (8.1.1, 127410, BioLegend), Ter119-APC/Cy7 (TER-119, 116223, BioLegend), CD45-BV605 (30-F11, 103140, BioLegend), CD106-AF647 (429, 105712, BioLegend), CD3 (145-2C11, 100304, BioLegend), CD4 (GK1.5, 100404, BioLegend), CD8a

(53-6.7, 100704, BioLegend), CD49b (DX5, 108904, BioLegend), CD90.2 (30-H12, 105304, BioLegend), CD19 (6D5, 115503, BioLegend), B220 (RA3-6B2, 103204, BioLegend), NK1.1 (PK136, 108704, BioLegend), TER119 (TER-119, 116204, BioLegend), CD11b (M1/70, 101204, BioLegend), CD11c (N418, 117304, BioLegend), Gr1 (RB6-8C5, 108404, BioLegend), CD16/32-BV711 (93, 101337, BioLegend), CD34-FITC (RAM34, 553733, BD Biosciences), CD48-AF700 (HM48-1, 103426, BioLegend), CD115-BV421 (AFS98, 135513, BioLegend), CD150-PerCP/Cy5.5 (TC15-12F12.2, 115922, BioLegend), c-kit-PE/Cy7 (2B8, 105814, BioLegend), Sca-1-BV605 (D7, 108133, BioLegend), Flk2-PE (A2F10, 12-1351-82, eBioscience), streptavidin-APC/Cy7 (405208, BioLegend), c-kit-PE/Cy7 (2B8, 105814, BioLegend), Sca-1-BV421 (D7, 108127, BioLegend), Flk2-PE (A2F10, 12-1351-82, eBioscience), CD48-AF700 (HM48-1, 103426, BioLegend), CD150-PerCP/Cy5.5 (TC15-12F12.2, 115922, BioLegend), CD34-FITC (RAM34, 553733, BD Biosciences), CD16/32-APC/Cy7 (93, 101327, BioLegend), CD115-PerCP/eFlour710 (AFS98, 46-1152-82, eBioscience), PE anti-biotin (1D4-C5, 409004, BioLegend), B220-PE (RA3-6B2, 103208, BioLegend), CD90.2-PE (53-2.1, 140308, BioLegend), CD49b-PE (DX5, 108908, BioLegend), CD103-PE (2E7, 121406, BioLegend), NK1.1-PE (PK136, 108708, BioLegend), Ter119-PE (TER-119, 116208, BioLegend), Ly6G-PE (1A8, 551461, BD Biosciences), CD11b-APC/Cy7 (M1/70, 101225, BioLegend), CD45-BV711 (30-F11, 103147, BioLegend), F4/80-PE/Cy7 (BM8, 123114, BioLegend), Ly6C-FITC (HK1.4, 128006, BioLegend), MHCII-BV605 (M5/114.15.2, 107639, BioLegend), Ly6G-FITC (1A8, 127605, BioLegend), CD11b-APC (M1/70, 101212, BioLegend), CD3-PE (17A2, 100206, BioLegend), CD90.2-PE (53-2.1, 140308, BioLegend), CD19-PE (1D3/CD19, 152408, BioLegend), B220-PE (RA3-6B2, 103208, BioLegend), F4/80-PE/Cy7 (BM8, 123114, BioLegend), Ly6C-BV605 (HK1.4, 128035, BioLegend), CD45-BV711 (30-F11, 103147, BioLegend), anti-mouse B220-PE/Cy7 (RA3-6B2, 103222, BioLegend), CD19-PE/Cy7 (6D5, 115520, BioLegend), CD3-PE (17A2, 100206, BioLegend), CD45-AF700 (30-F11, 103127, BioLegend), Ly6C-BV605 (HK1.4, 128035, BioLegend), Ly6G-FITC (1A8, 127605, BioLegend), CD11b-APC (M1/70, 101212, BioLegend), NK1.1-APC/Cy7 (PK136, 108730, BioLegend), CD115-BV421 (AFS98, 135513, BioLegend), anti-BrdU-APC (51-23619L, BD Biosciences), Ki-67-eFluor660 (SolA15, 50-5698-82, eBioscience), CD11b-PE (M1/70, 101208, BioLegend), CD3-PerCP/Cy5.5 (145-2C11, 551163, BD Biosciences), CD8a-BV711 (53-6.7, 100748, BioLegend), CD4-AF700 (GK1.5, 100430, BioLegend), CD45.2-APC (104, 109814, BioLegend), CD45.1-PE/Cy7 (A20, 560578, BD Biosciences), CD19-APC/Cy7 (6D5, 115529, BioLegend), B220-APC/Cy7 (RA3-6B2, 103224, BioLegend), NK1.1-APC/Cy7 (PK136, 108724, BioLegend).

Antibodies for immunohistochemistry, immunofluorescence, and intravital microscopy:
Anti-mouse Collagen type I (AB765P, MilliporeSigma), Collagen type III (ab7778, Abcam), Collagen type IV (AB756P, MilliporeSigma), Endomucin (AF4666, Novus Biologicals), Laminin (ab11575, Abcam), CD11b (M1/70, 550282, BD Biosciences), CD31 (MEC13.3, 557355, BD Biosciences), alpha smooth muscle Actin (ab5694, Abcam), Sca-1-PE (D7, 108108, BioLegend), CD31-PE (MEC13.3, 102508, BioLegend), CD11b-APC (M1/70, 101212, BioLegend), Sca-1-AF647 (D7, 108118, BioLegend).

Antibody used for in vivo experiments:
InVivoMAb anti-mouse Vegfr2 (clone DC101, BE0060, BioXCell).

Validation

All antibodies used for flow cytometry, immunohistochemistry, and immunofluorescence were previously validated for the respective application by the distributor. Antibodies used for intravital microscopy were validated for this application by the investigators in preceding studies (Vandoore K, Rohde D et al. Circ Res 2018; 123 (4): 415-427). Validation of InVivoMAb anti-mouse Vegfr2 (clone DC101, BE0060, BioXCell) was performed by Hooper AT et al. (Hooper AT et al. Cell Stem Cell 2009; 4 (3): 263-274).

Animals and other organisms

Policy information about studies involving animals; ARRIVE guidelines recommended for reporting animal research

Laboratory animals

The following mouse strains were used: Wild type C57BL/6J, Apoetm1Unc, BPN/3J, BPH/2J, B6.Cg-Gt(ROSA)26Sortm6(CAG-ZsGreen1)Hze/J, B6.Cg-Commd10Tg(Vav1-icre)A2Kio/J, Kdrtm2Sato/J (all purchased from The Jackson Laboratory, Bar Harbor, ME, USA); Cdh5(PAC)-CreERT2 (purchased from Taconic, Rensselaer, NY, USA), Vcantm1.1Hwat (provided by H. Watanabe, Institute for Molecular Science of Medicine, Aichi Medical University, Aichi, Japan), Il6tm1.1Jho (provided by J. Hidalgo, Autonomous University of Barcelona, Barcelona, Spain), Aplntm1.1(cre/ERT2)Bzsh (provided by Bin Zhou, Shanghai Institutes for Biological Sciences, Chinese Academy of Sciences, Shanghai, China). Animals were sacrificed for experiments at 12 to 30 weeks of age. Where appropriate, age- and sex-matched mice were used for experiments.

Wild animals

This study did not involve wild animals.

Field-collected samples

This study did not involve field-collected samples.

Ethics oversight

Animal protocols were approved by the Institutional Animal Care and Use Committee (IACUC) at Massachusetts General Hospital. All animal experiments were performed in compliance with relevant ethical regulations and all efforts were made to avoid suffering of animals.

Note that full information on the approval of the study protocol must also be provided in the manuscript.

Human research participants

Policy information about studies involving human research participants

Population characteristics

Arterial hypertension patients had previously diagnosed arterial hypertension (based on office systolic blood pressure ≥140 mmHg and/or diastolic blood pressure ≥90 mmHg in repeated measurements) and chronic anti-hypertensive drug treatment. 83.33% of patients in this group had systolic blood pressure values ≥140 mmHg and/or diastolic blood pressure values ≥90 mmHg at the time of sample collection. Atherosclerosis and arterial hypertension patients had previously diagnosed coronary artery disease, cerebrovascular disease and/or peripheral artery disease combined with arterial hypertension. Sample collection in patients with acute myocardial infarction was performed between day 3 and day 7 after diagnosis of ST-segment elevation myocardial infarction (STEMI). Percutaneous coronary intervention (PCI) was performed in all patients on the day of onset of clinical symptoms. Healthy control subjects had no medical history for any of the aforementioned diagnoses and no chronic pharmacological treatment. Additional bone marrow samples from healthy controls were purchased from Stemcell Technologies (Cambridge, MA, USA) and HemaCare (Los Angeles, CA, USA). Age distribution in controls and patient cohorts

	was as follows (displayed as mean±SD years): control 55.3±13.5, hypertension 69.8±15.9, atherosclerosis+hypertension 64.75 ±7.4, myocardial infarction 61.1±11.1.
Recruitment	Patient recruitment was performed at University Hospital Germans Trias i Pujol, Amsterdam University Medical Center, and Massachusetts General Hospital. Patients were eligible for enrollment with a) one or more of the following previously diagnosed medical conditions or b) non of the following diagnoses and no chronic pharmacological treatment: 1) arterial hypertension (with chronic anti-hypertensive drug treatment), 2) atherosclerosis (defined as coronary artery disease, cerebrovascular disease, and/or peripheral artery disease), 3) acute ST-segment elevation myocardial infarction (STEMI).
Ethics oversight	Study protocols were approved by the Institutional Review Boards of University Hospital Germans Trias i Pujol (ICOR-2017-06, Ref. PI-17-176), Amsterdam University Medical Center (NTR166, ISRCTN95796863), and Massachusetts General Hospital (2013P002438, 2018P001465).

Note that full information on the approval of the study protocol must also be provided in the manuscript.

Flow Cytometry

Plots

Confirm that:

☒ The axis labels state the marker and fluorochrome used (e.g. CD4-FITC).

☒ The axis scales are clearly visible. Include numbers along axes only for bottom left plot of group (a 'group' is an analysis of identical markers).

☒ All plots are contour plots with outliers or pseudocolor plots.

☒ A numerical value for number of cells or percentage (with statistics) is provided.

Methodology

Sample preparation	Human bone marrow:<br>Human bone marrow samples were immersed in ice-cold sterile PBS with 0.5% bovine serum albumin and passed through a 40µm cell strainer. Following centrifugation, the cell pellet was resuspended in 70% Dulbecco's Modified Eagle's Medium without phenol red (D1145, Sigma-Aldrich, St. Louis, MO, USA), 20% fetal calf serum, and 10% DMSO, transferred to cryovials and stored in liquid nitrogen. For flow cytometry, cells were thawed at 37°C, washed in PBS, and stained with the respective antibodies.<br><br>Mouse tissue:<br>To assess bone marrow hematopoietic cells, mice were anesthetized and flushed with 20mL PBS in order to remove intravascular blood. Bones were dissected and the bone marrow was flushed out with FACS buffer (PBS containing 0.5% bovine serum albumin). A single-cell suspension was created by passing the bone marrow through a 40µm cell strainer. To investigate hematopoietic cells in the spleen, spleen tissue was minced and plunged through a 40µm cell strainer, before red blood cell lysis was performed with RBC lysis buffer (420301, BioLegend). Peripheral blood was collected by retro-orbital bleeding using heparinized capillary tubes (420316, BD, Sparks, MD, USA) and red blood cells were lysed in RBC lysis buffer (BioLegend). For bone marrow stroma cells, bone marrow was flushed out and enzymatically digested in Dulbecco's Modified Eagle's Medium (DMEM) without phenol red (D1145, Sigma-Aldrich) containing 1mg/mL Collagenase Type 4 (LS004188, Worthington Biochemical Corporation, Lakewood Township, NJ, USA) and 2mg/mL Dispase II (D4693, Sigma-Aldrich) for 30min at 37°C with gentle agitation. Spleen tissue was minced and subjected to the same digestion protocol for the analysis of stroma cells. Skeletal muscles were dissected, minced into small pieces and enzymatically digested in PBS containing 450U/mL Collagenase Type 1 (C0130), 125U/mL Collagenase Type 11 (C7657), 60U/mL DNase I (D5319) and 60U/mL hyaluronidase (H3506, all purchased from Sigma-Aldrich) for 60min at 37°C under constant agitation.
Instrument	Flow cytometry data were acquired on an LSRII flow cytometer (BD Biosciences, San Jose, CA, USA).
Software	FACS Diva 6.1 software (BD Biosciences, San Jose, CA, USA) was used for data collection. Data were analyzed with FlowJo 10 software (BD, Franklin Lakes, NJ, USA).
Cell population abundance	Purity of sorted cells was >95% as assessed by confirmatory flow cytometry.

Gating strategy

Human bone marrow:
After pregating on single live cells, phenotypic hematopoietic stem cells (HSC) were identified as lineage- CD34+ CD38- CD45RA(low) CD90+, common myeloid progenitors (CMP) as lineage- CD34+ CD38(int) CD45RA- CD123(int) and granulocyte/ monocyte progenitors (GMP) as lineage- CD34+ CD38(int) CD45RA+ CD123(int).

Mouse tissue:
All cell populations were pregated on viable and single cells. Bone marrow and spleen EC were identified as Ter119- CD41- CD45- CD31high Sca-1+. Arteriolar EC were identified as Ter119- CD41- CD45- CD31high Sca-1+ Pdpn- and sinusoidal EC as Ter119- CD41- CD45- CD31high Sca-1+ Pdpn+. Bone marrow MSC were identified as Ter119- CD41- CD45- CD31- Sca-1- CD106+, and fibroblasts as Ter119- CD41- CD45- CD31- CD106- Sca-1+. Skeletal muscle endothelial cells were identified as CD45- CD31+. LSK were identified as Lin- c-kit+ Sca-1+ and SLAM-LSK as Lin- c-kit+ Sca-1+ CD150+ CD48-. Common myeloid progenitors (CMP) were identified as Lin- c-kit+ Sca-1- CD16/32int CD34+ and granulocyte/monocyte progenitors (GMP) as Lin- c-kit+ Sca-1- CD16/32high CD34+. Monocytes were identified as CD45+ NK1.1- CD3- CD19- B220- CD11b+ SSC-Alow CD115+ Ly6G- and neutrophils as CD45+ NK1.1- CD3- CD19- B220- CD11b+ SSC-Alow CD115- Ly6G+.

☒ Tick this box to confirm that a figure exemplifying the gating strategy is provided in the Supplementary Information.
